# Recombination between fragile regions associated with chromosomal rearrangements in glioblastoma can be mediated by RAGs

**DOI:** 10.1016/j.isci.2025.113815

**Published:** 2025-10-21

**Authors:** Amita Paranjape, Susmita Kumari, Lipsa Rani Sahu, Amrita Mondal, Swapna Kunhiraman, Arun Sharma M, Namrata M. Nilavar, Bibha Choudhary, Sathees C. Raghavan

**Affiliations:** 1 Department of Biochemistry, Indian Institute of Science, Bangalore 560012, India; 2 Institute of Bioinformatics and Applied Biotechnology, Electronics City, Bangalore 560100, India

**Keywords:** chromosome organization, molecular interaction, cancer, transcriptomics

## Abstract

The RAGs, comprising RAG1 and RAG2, catalyze V(D)J recombination by recognizing recombination-signal sequences (RSS). Glioblastoma, the aggressive brain cancer, has many oncogenic chromosomal alterations; however, the mechanism of their generation is largely unknown. Here, we report that RAGs are expressed in human glioblastoma cells at transcript and protein levels. RNA-seq data analysis confirmed the expression of RAGs in the majority of patients with glioma. Analysis of patient breakpoint sequences reveals cryptic RSS in regions undergoing rearrangements. Biochemical studies demonstrate that RAGs can bind and cleave cryptic RSS in fragile regions (AMY1B, CAMK2D, RN7SKP123-MTF2, DIPK1A, IRX5-IRX6), albeit at lower efficiency. Recombination assay using episomes harboring the fragile regions showed aberrant recombination in these regions, and the efficiency was significantly reduced in RAG1 ablated cells. Finally, we recapitulate the glioblastoma associated AMY1B and RN7SKP123-MTF2 chromosomal rearrangement using an extrachromosomal assay. Thus, the present study provides mechanistic insights into the generation of chromosomal aberrations associated with glioblastoma.

## Highlights

• RAGs are expressed and form a functional complex in glioblastoma cells and patients
• RAGs bind and cleave cryptic RSS in the fragile genomic regions from patients with glioma
• Recombination at fragile regions associated with glioblastoma is dependent on RAGs
• The study provides mechanistic insights into chromosomal aberrations in glioblastoma

## Graphical abstract

**Figure:**
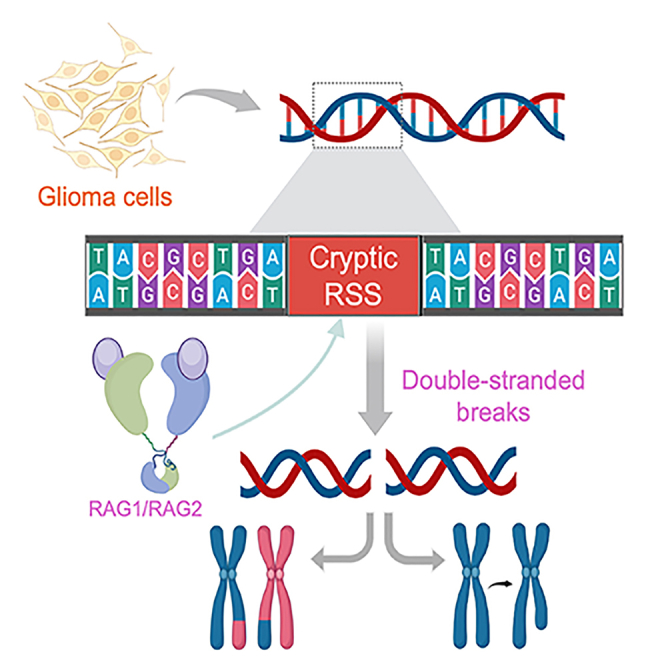

## Introduction

Among various cancers, brain cancer is considered one of the most lethal ones. There was an increase in the global burden of brain cancer by over 3,00,000, and morbidity by 2,50,000 (GLOBOCON 2020; https://gco.iarc.fr). Based on aggressiveness, IDH (isocitrate dehydrogenase) mutation status, and genomic alteration (such as 1p19q co-deletion), brain cancer is classified into oligodendroglioma, anaplastic and diffuse astrocytoma, and glioblastoma by the WHO in 2016.^1^ Glioblastoma is a deadly brain cancer, accounting for 70% of gliomas. The average life span of patients since their diagnosis is less than a year in most cases, which makes it the most detrimental.^2^^,^^3^^,^^4^^,^^5^

Glioblastoma multiforme (GBM) has various oncogenic alterations, such as in-frame deletion events generating epidermal growth factor receptor variant III (EGFRvIII), heterozygous deletion of NF-κB inhibitor alpha (NFKBIA), amplification/mutation of RTK (either EGFR or PDGFRA), chromosomal translocation generating FGFR1-TACC1 and FGFR3-FGFR3, fusion events generating EGFR-SEPT14 and EGFR-PSPH, mutation/deletion of TP53 and PTEN, CDKN2A or CDKN2B deletion, loss of chromosome 10 and gain of chromosome 7, and so forth.^6^^,^^7^^,^^8^^,^^9^^,^^10^^,^^11^^,^^12^^,^^13^^,^^14^^,^^15^^,^^16^

RAG complex, consisting of RAG1 and RAG2, is the endonuclease involved in V(D)J recombination.^17^^,^^18^^,^^19^^,^^20^ RAGs recognize the sequence known as the recombination signal sequence (RSS), consisting of a conserved heptamer (CACAGTG), and a nonamer (ACAAAAACC) separated by a nonconserved spacer sequence of 12 or 23 nt, based on which it is called a 12RSS or 23RSS.^21^^,^^22^^,^^23^ RAGs bind to RSS, introducing a nick at 5′ of the heptamer, resulting in a free hydroxyl group that undergoes a transesterification reaction on the opposite strand, forming a hairpin. Two such hairpins formed are further processed by DNA-PKcs/Artemis endonuclease, followed by repair through non-homologous end-joining (NHEJ), forming a joint that codes for a variable region of antibody or TCR.^24^^,^^25^^,^^26^^,^^27^

Besides its physiological function, RAGs can cleave cryptic RSS that resembles standard RSS with a few nucleotide variations.^28^^,^^29^^,^^30^^,^^31^ RAGs can also recognize non-B DNA structures such as heterologous loops, bubbles, heteroduplex DNA of hairpins, cruciform, G-quadruplexes, and so forth, and nick at single-/double-strand DNA transition leading to double-strand breaks.^32^^,^^33^^,^^34^ Excised signal circles (ESC), created during RAG action, are either lost in subsequent cell division or act as transposons.^35^^,^^36^^,^^37^^,^^38^ This indicates that, in addition to their physiological function, RAGs can act as a key player during the generation of chromosomal rearrangements.

RAGs are generally expressed in B and T cells, although the occurrence of RAGs outside the lymphoid system has been reported. The Baltimore lab observed the presence of RAG1 transcript in the murine brain;^39^ however, its relevance was not established. Reduced DNA damage response and cellular fitness were observed in RAG-deficient NK cells.^40^ RAG2 has been reported to be necessary for the normal development of plasmacytoid dendritic cells (pDCs).^41^ The expression of RAG2 has been reported in various tumor cell lines such as Ewing’s sarcoma cell line A673, osteosarcoma cell line U-2 OS, PBMC, 203-glioma, C-1300 neuroblastoma, and B-16 melanoma and fibroblast HT1080.^42^^,^^43^^,^^44^^,^^45^^,^^46^

In the present study, we report that RAG1 and RAG2 are differentially expressed in various glioblastoma cell lines studied and can bind and catalyze recombination between canonical RSS. RNA seq data analysis showed detectable expression of RAG1 and RAG2 in most of the patients. *In silico* analysis of the COSMIC database revealed the presence of cryptic RSS near breakpoints that undergo chromosomal rearrangements in brain cancer, and purified RAGs could bind and cleave at the cryptic RSS. Extra-chromosomal recombination assay showed that recombination can take place between the fragile regions and standard RSS within glioma cells, albeit at low efficiency, and was dependent on RAGs.

## Results

### RAG1 and RAG2 are expressed in patients with glioblastoma and cell lines

Generally, it is well established that RAG1 and RAG2 are expressed in lymphoid cells, particularly during the early developmental stages of B and T cells. Although it has been demonstrated that RAG1 transcripts are present in brain cells, there have not been many studies to evaluate the role asserted to such an aberrant expression of RAGs.^39^ Previous reports indicated that many chromosomal rearrangements seen in patients with glioma exhibited breakpoint junctions, characteristic of RAG-mediated recombination.^47^ Therefore, we were interested in investigating the role of RAGs in brain cancer.

**Figure 1:**
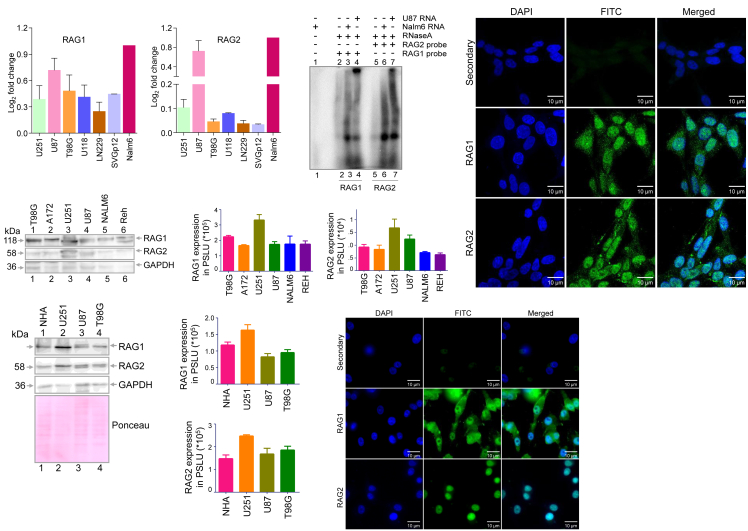
Expression analysis of RAG1 and RAG2 in different glioma cell lines (A and B) Bar graph depicts the quantitative expression of *RAG1* (A) and *RAG2* (B) through qRT-PCR (*n* = 3) in different glioblastoma cell lines (U251, U87, T98G, U118, LN229), an immortalized astrocyte cell line (SVGp12), and a pre-B cell leukemia cell line (Nalm6). qRT-PCR was performed using cDNA prepared from different cell lines after normalization with 18S rRNA as an internal control. Error bar represents mean ± SEM. (C) RNaseA protection assay profile showing comparative *RAG1* and *RAG2* expression at the RNA level in a glioblastoma cell line, U87, and a leukemic cell line, Nalm6. (D) Western blotting shows the expression of RAG1 and RAG2 in different GBM cell lines (T98G, A172, U251, U87) and leukemia cell lines (Nalm6, Reh). GAPDH was used as a loading control. (E and F) Bar graph shows RAG1 (E) and RAG2 (F) quantification in cell lines, T98G, A172, U251, U87, Nalm6, and Reh after normalization with GAPDH expression. Error bar represents mean ± SEM. (G) Western blotting shows the expression of RAG1 and RAG2 in an immortalized astrocyte cell line (NHA) compared to that of GBM cell line (U251, U87, and T98G). (H and I) Bar graph shows the quantification of RAG1 (H) and RAG2 (I) in the immortalized astrocyte cell line compared to different GBM cell lines after normalization with ponceau stained blot. Error bar represents mean ± SEM. (J and K) Immunofluorescence study to evaluate the intracellular localization of RAG1 and RAG2 in GBM cell lines, U87 (J) and T98G (K), using anti-RAG1 and RAG2. In both panels, the nucleus is stained by DAPI, and FITC is conjugated with respective secondary antibodies. Scale bar represents 10 μm.

Firstly, we assessed the expression levels of RAG1 and RAG2 across a series of glioma cell lines, U251, U87, T98G, U118, and LN229. Total RNA was isolated from the glioma cells along with immortalized astrocyte cell lines, SVGp12 and Nalm6 (B-cell precursor leukemia), using TRI reagent, and was used to prepare cDNA. Genomic DNA contamination in cDNA was ruled out using reactions without reverse transcriptase. cDNA was normalized using 18S rRNA as an internal control. Real-time PCR was performed to evaluate the expression of RAG1 and RAG2 in these cell lines. Results showed that the RAG1 expression was high in U87, T98G, U251, and U118 cells compared to LN229 (Figure 1A). Interestingly, SVGp12, the transformed astrocyte cell line, also showed moderate RAG1 expression. In case of RAG2, expression was high in U87, followed by U251, whereas low RAG2 expression was observed in case of T98G, LN229, and U118 (Figure 1B). In the case of SVGp12, RAG2 expression was significantly low. This suggests that both RAG1 and RAG2 are differentially expressed across glioma cell lines. Besides, RAG1 and RAG2 expression were lower in glioma cell lines compared to the Nalm6, a pre-B cell line.

Further, expression at the transcript level was assessed using RNase protection assay, in which [^α−32^P] ATP radiolabeled probe derived from RAG1 and RAG2 was hybridized with total RNA (∼20 μg) isolated from U87 and Nalm6 cell lines. Following hybridization, reactions were subjected to RNase A digestion, purified, and resolved on 1% agarose gel. Results showed that the expression of *RAG1* at the RNA level in U87 was significantly lower than that of Nalm6, while *RAG2* expression was comparable in both cell lines (Figure 1C). These results suggest that both RAG1 and RAG2 transcripts were present in glioma cells, and the expression of RAG2 was high in U87 compared to RAG1.

Further expressions of RAG1 and RAG2 were investigated at the protein level by Western blotting analysis. To do this, cell extract was prepared from glioma cell lines U251, U87, T98G, and A172. The proteins were equalized on an SDS-PAGE and were used for western blotting. Pre-B leukemic cell lines, Nalm6 and Reh, served as positive controls for the expression of RAGs. Significant expression of RAG1 was seen in all human glioma cell lines. Interestingly, the highest expression was seen in U251 and T98G. Notably, at the protein level, RAG1 and RAG2 expression in the GBM cell line was comparable to that of Nalm6 and Reh cells (Figures 1D–1F). The lower molecular weight bands observed in some of the cell lines could be due to ubiquitylated or proteolytically processed forms of RAG1 and RAG2. Such processed forms were reported previously upon the ectopic expression of RAG1 and RAG2 in human cells.^48^ The normal human immortalized astrocyte cell line (NHA) was used to compare RAG expression in glioma cell lines (Figures 1G–1I). Importantly, western blotting using anti-RAG1/RAG2 revealed a moderate increase in the expression of RAG1 and RAG2 in U251 cells, whereas U87 and T98G showed differential expression compared to NHA cells (Figures 1G–1I). These results suggest that RAG1 and RAG2 are expressed in glioma cells, although there is variation in the levels of expression.

Further immunofluorescence (IF) analysis was performed to evaluate the expression of RAG1 and RAG2 at the intracellular level in two glioma cell lines. IF studies using anti-RAG1 and anti-RAG2 revealed that the expression of RAG1 and RAG2 was mainly confined to the nucleus in U87MG (Figures 1J and S1A) and T98G (Figures 1K and S1B) compared to secondary antibody control. To further confirm the specificity of RAG1 and RAG2 antibodies, we performed IF in U87 wild type and RAG1 knock out cells. Results revealed the robust expression of both RAG1 and RAG2 in the case of U87 wild type cells, however, limited the expression of both proteins was observed in the case of RAG1 knock-out cells. These results further confirm the specific binding of the antibodies to their respective targets, RAG1 and RAG2 (Figures S1C and S1D). In summary, using various molecular biology techniques, we show that RAG1 and RAG2 proteins are expressed in different glioma cell lines, although the overall expression level was lower than the pre-B cell lines.

**Figure 2:**
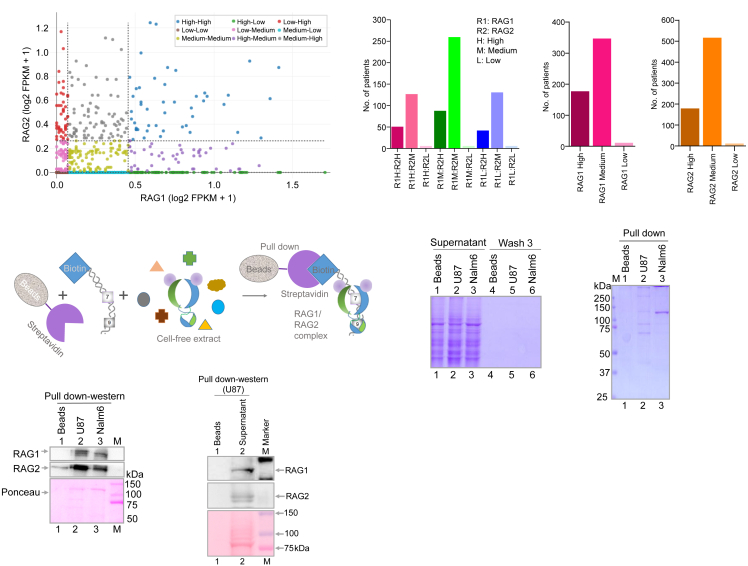
Analysis of RAG1 and RAG2 expression based on RNA seq data from patients with glioblastoma and assessment of intracellular binding of RAG1 and RAG2 to 12RSS within glioblastoma cells (A) A bi-variate expression scatterplot shows the RAG1 and RAG2 expression level in different patients with glioblastoma (*n* = 693). Samples were plotted based on log_2_-transformed FPKM values for RAG1 (X axis) and RAG2 (Y axis). Dashed vertical and horizontal lines represent the 25^th^ and 75^th^ percentile thresholds used to classify samples into low, medium, and high expression groups for each gene. Samples are color-coded according to their combined RAG1 and RAG2 expression. (B) Bar graph shows the number of patients with glioblastoma in each of the combined RAG1 and RAG2 expression groups. Expression levels of RAG1 (R1) and RAG2 (R2) were categorized as High (H), Medium (M), or Low (L) based on 25^th^ and 75^th^ percentile thresholds. (C and D) Bar graphs depicts the distribution of patients with glioblastoma based on RAG1 (C) or RAG2 (D) expression alone, categorized into high, medium, or low expression groups. (E) Schematic of the experimental procedure used for the streptavidin pull-down assay. The cell-free extract was prepared from the glioblastoma cell line, U87, and was incubated with 12RSS oligomeric DNA substrate conjugated with biotin. Pulldown assay was performed with streptavidin-conjugated beads. (F) PAGE profile shows supernatant obtained from samples, beads alone (without incubation with DNA substrate), U87 extract, and Nalm6 extract. (G) Gel profile shows the corresponding pulldown obtained from the same experiment when incubated with U87 and Nalm6 cell lines. (H) Western blotting of pulldown samples from U87 and Nalm6 cell lines for RAG1 and RAG2. (I) Cell-free extract from the U87 glioblastoma cell line was incubated with a biotin-conjugated oligomeric DNA substrate that does not contain a conserved heptamer and nonamer sequence. Subsequently, pulldown assay was performed using streptavidin-conjugated beads, and the pulldown samples were analyzed by western blotting for RAG1 and RAG2 proteins.

To investigate the expression pattern of RAG1 and RAG2 in patients with glioblastoma, we analyzed RNA-seq data of 693 patient samples obtained from the Chinese Glioma Genome Atlas (CGGA) database. Scatterplot analysis was performed to compare the expression levels in each patient (Figure 2A). Based on the 25^th^ and 75^th^ percentile thresholds for both RAG1 and RAG2 expression, patients were stratified into nine distinct subgroups reflecting all possible combinations of low, medium, and high expression levels for RAG1 and RAG2. Patients falling below the 25^th^ percentile were categorized as “Low,” those between the 25^th^ and 75^th^ percentiles as “Medium,” and those above the 75^th^ percentile as “High.” Results revealed a heterogeneous pattern of RAG1 and RAG2 expression in gliomas, with a strong bias toward high and mid-level expression of both genes (Figure 1A).

We quantified the number of patients in each of the combinatorial RAG1 and RAG2 expression categories. Majority of the samples exhibited medium expression of both RAG1 and RAG2 followed by RAG1-High/RAG2-Medium (R1H/R2M) and RAG1-Low/RAG2-Medium (R1L/R2M) expression level (Figure 2B). The least populated categories involved low RAG1 expression combined with low or high RAG2, or vice versa, supporting the observation that the low expression of either gene is rare (Figure 2B).

We also looked at the individual expression profiles of RAG1 and RAG2. A large number of patients with glioblastoma showed medium expression of RAG1 (∼350 patients), while high RAG1 expression was observed in ∼180 samples (Figure 2C). In contrast, RAG2 expression was predominantly medium (∼500 patients), with fewer samples in the high-expression group and no patients in the low-expression group (Figure 2D). This indicates that RAG2 expression is consistently maintained at moderate to high levels in glioblastoma, while RAG1 exhibits both high and medium levels in the majority of the patients. Taken together, these results highlight a non-random and coordinated expression pattern of RAG1 and RAG2 in glioblastoma, with very few samples displaying low levels of either gene.

### RAG1 and RAG2 interact and bind to canonical 12recombination signal sequence DNA within glioma cells

We were interested in determining if RAG1 and RAG2 interact with each other such as lymphoid cells and can form a complex within the glioma cells. Besides, such a RAG complex, if present within glioma cells, can bind specifically to the canonical 12RSS DNA. To test this, we employed the “Streptavidin-agarose pull-down assay” to capture and isolate the protein-DNA interactions (Figure 2E). The cell-free extract from U87 (30 μg) was incubated with 5′-biotinylated double-stranded canonical 12RSS DNA or 5′-biotinylated double-stranded DNA, which does not contain conserved heptamer and nonamer (as a control), DNA probes in the presence of streptavidin-agarose beads. The proteins bound to biotinylated DNA were pulled down, washed, and resolved on SDS-PAGE and subjected to western blotting analysis. CBB profile exhibited a uniform protein loading between extracts incubated with beads alone or beads and biotinylated 12 RSS, when supernatant was loaded (Figure 2F, lanes 1–3). Further, as expected, the wash fraction did not show any protein in all three cases (Figure 2F, lanes 4–6). Besides, no bands were seen on SDS-PAGE when pull-down fractions were resolved following the incubation of beads alone with the cell-free extracts (Figure 2G, lane 1). In contrast, distinguishable bands were observed when biotinylated DNA harboring 12RSS was incubated with the extracts (Figure 2G, lanes 2–3). Western blotting of the pull-down samples showed that both RAG1 and RAG2 were present and remained as a complex within the glioblastoma cell line, U87, as compared to the positive control, Nalm6 (Figure 2H). However, no RAG1 or RAG2 was detected in the pull-down samples using the control DNA substrate lacking the 12RSS sequence (Figure 2I), further confirming the specific binding of the RAG complex to the 12RSS substrate in the U87 glioma cell line. In the case of RAG2, a distinct lower molecular weight band was observed, which could be a variant (Figure 2I). Thus, our results suggest that RAG1 and RAG2 present in glioblastoma cells can interact and bind to DNA containing 12RSS.

### RAGs present in glioma cells can catalyze recombination within the cells

**Figure 3:**
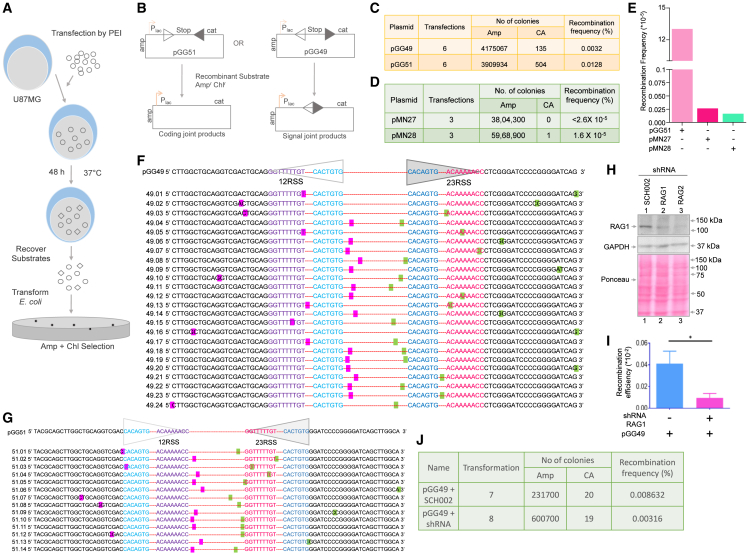
Evaluation of RAG activity in glioma cells based on recombination between 12 and 23 signals (A) Schematic showing extrachromosomal V(D)J recombinase assay in glioblastoma cell line, U87. pGG49 and pGG51 were used for evaluating the efficiency of recombination catalyzed by RAGs present in U87 cells. (B) Cartoon indicates recombination constructs pGG51 and pGG49, which contain consensus 12 signal (open triangle) and 23 signal (closed triangle). Recombination between signal sequences could lead to either a coding joint or a signal joint, resulting in the deletion of the in-between “Stop” sequence. U87 cells were transfected with either pGG51 or pGG49 construct. 48 h post-transfection, minichromosomes were harvested and transformed into *E. coli*. to detect recombinants on ampicillin and chloramphenicol (CA) LB agar plates. “*cat”* denotes the chloramphenicol acetyltransferase gene, and “Stop” denotes the prokaryotic transcription terminator. The *E. coli lac* promoter is denoted as *P*_*lac*_. (C) Table shows a summary of total ampicillin (Amp) resistant and chloramphenicol-ampicillin (CA) resistant colonies obtained, along with the recombination frequency of pGG49 and pGG51. The data shown is derived from multiple transfections (*n* = 6) followed by transformations. (D) pMN27 and pMN28, containing only the unpaired 23RSS and 12RSS, respectively, were transfected into the U87 cell line, and the recombination was evaluated following transformation into *E. coli*. The table represents the total number of ampicillin resistant and chloramphenicol-ampicillin resistant colonies, along with the recombination frequencies observed for pMN27 and pMN28. (E) The recombination frequency was calculated for control episomes, pMN27 and pMN28, and presented as a bar graph in comparison with pGG51. (F and G) Sequencing results show recombinant junctions obtained from pGG49 (F) and pGG51 (G) following transfections in the U87 cell line. The open triangle represents the 12 RSS, while the closed triangle represents 23 RSS; the top sequence is of parental plasmid, whereas the rest are from the recombinant clones. Pink and green highlight indicates the sequence at the recombination junction. The 12 RSS heptamer is shown in sky blue, and the nonamers are in purple, whereas in the 23 RSS, the heptamers are in blue and the nonamers are in pink. (H) Western blotting shows the efficiency of knockdown of RAG1 and RAG2 following the transfection of shRNA (RAG1-D10 and RAG2-D4). SCH002 is a scrambled shRNA. (I and J) Bar graph shows the recombination efficiency of pGG49 upon RAG1 knockdown (*n* = 3). The error bar was calculated as mean ± SEM (∗*p* < 0.05, “ns” nonsignificant) (I). Table showing recombination frequency following the transfection of signal joint construct pGG49 upon using 2 different shRNAs for RAG1 knockdown (J). As a control, pGG49 was co-transfected with an equal amount of SCH002 (scrambled plasmid).

It is well established that RAGs expressed in B and T cells catalyze V(D)J recombination and are essential for generating diversity of immunoglobulin and T cell receptors. Since RAG1 and RAG2 expression was seen in glioblastoma cell lines, we wondered whether the RAGs can bind to canonical 12RSS DNA and catalyze the V(D)J recombination, as this may explain several genomic rearrangements seen during glioblastoma. To investigate this, we performed an extrachromosomal V(D)J recombination assay to evaluate the recombination potential in U87 cells. For this, pGG49 and pGG51 episomes were used, in which signal sequences were cloned in such a way that RAG-mediated recombination can lead to either signal joint or coding joint products. Such recombination can provide chloramphenicol resistance to the recombined episome upon transformation into *E. coli* (Figures 3A and 3B). Recombination frequency was assessed based on the selection of Amp and Chl-Amp (CA) in *E. coli* following the transfection of either pGG49 or pGG51 in U87 cells. Results revealed several CA-resistant colonies following the transformation of transfection products from glioblastoma cells in the case of pGG49 (135 colonies) and pGG51 (504 colonies) (Figure 3C). Recombination frequency determined in the case of pGG49 was 0.0032% in U87 glioblastoma cells, while it was 0.0128% for pGG51 (Figure 3C). Therefore, a ∼4-fold increase in coding joint formation was observed compared to signal joint formation. This indicates that RAGs present in the glioma cells are active and can facilitate recombination between the 12 and 23RSS signals. To analyze the background recombination frequency, we have transfected the episomes containing either 12RSS (pMN28) or 23RSS (pMN27) in the pGG51 plasmid backbone, as negative controls. Upon transformation, we did not observe any recombinants in the case of pMN27 episomal substrate, whereas only one recombinant was observed in the case of pMN28 (Figures 3D and 3E). The recombination frequency in U87 cells was approximately ∼500-fold and 800-fold lower for the pMN27 and pMN28, respectively, compared to the pGG51 (Figures 3D and 3E). These results suggest that recombination in U87 cells occurred specifically when both 12RSS and 23RSS sequences were present.

The identity of the recombinants obtained on the CA plate was further assessed following plasmid DNA isolation and restriction endonuclease digestion. The EcoRI digestion of recombinants from pGG49 transfected samples resulted in a diagnostic band of 405 bp for pGG49 and 328 bp for pGG51 in the case of recombinants, while an unrecombined episome would have generated a band of 658 bp (Figure S2A). Restriction digestion analysis showed that most CA-positive recombinants were positive upon digestion. However, the size of the characteristic band due to recombination was not an exact match (405 bp for pGG49 and 328 bp for pGG51) (Figures S2B–S2D). Therefore, DNA sequencing of the recombinant junctions was performed using primers SCR21 and GHG1. In the case of pGG49, the results confirmed recombination between 12 and 23RSS in most of the clones sequenced (Figure 3F). However, breakpoints did not map strictly at the heptamer of 12RSS or 23RSS, in the majority of recombinants derived from pGG51 (Figures 3G and S2E). It is possible that, unlike lymphoid cells, glioma cells possess additional nuclease action or lack protection from nucleases, resulting in the observed aberrant recombination. The operation of aberrant DNA repair pathways, such as MMEJ, could also be a contributing factor. Considering that several breakpoints were not present at the heptamer of the RSS, we were interested in testing whether RAGs were indeed responsible for the observed recombination. A recombination assay was performed following the knockdown of RAG1 and RAG2 using shRNA (RAG1D-10, RAG2-D4) in the U87 cell line to evaluate this. Western blot results revealed efficient knockdown of RAG1 and RAG2 compared to scrambled control (Figure 3H). The recombination assay results showed a significant reduction in recombination frequency following shRNA-mediated knockdown of RAG1 when the signal joint formation was analyzed (pGG49) (Figures 3I and 3J). These results suggest that observed recombination is due to RAG activity in glioblastoma cells.

### Cryptic recombination-signal sequences are present adjacent to breakpoint regions of patients with brain cancer and purified RAGs bind and cleave at cryptic recombination-signal sequences from such fragile regions

Bioinformatic analysis of high-grade glioma patient breakpoints retrieved from the COSMIC (Catalog of Somatic Mutations in Cancer) database (https://cancer.sanger.ac.uk) revealed the presence of cryptic RSSs in chromosomal rearrangements.^49^ We further narrowed down the search for breakpoint clusters where the distance between 2 breakpoints is ≤ 100 bp and 5 breakpoint clusters were selected from it for the study (Figures S3A–S3E).

**Figure 4:**
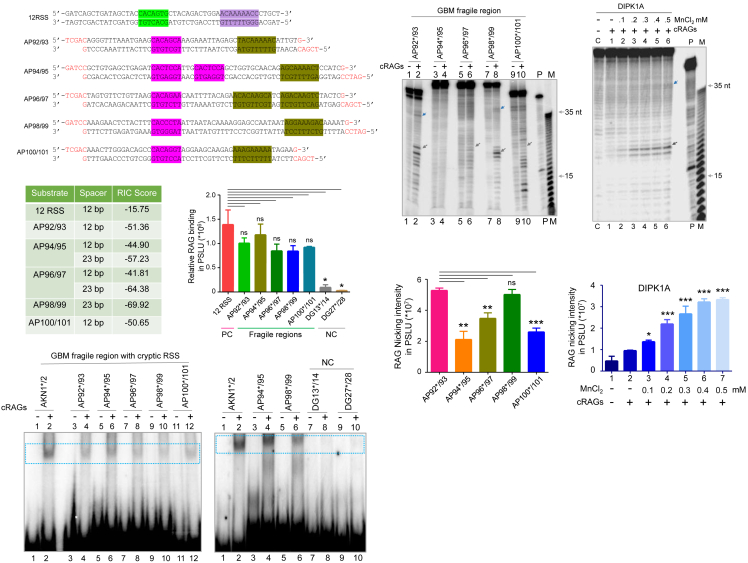
Evaluation of RAG-binding and nicking efficiency at fragile regions derived from brain cancer (A) Sequence of cryptic RSS substrates derived from GBM fragile regions. Substrates used were from breakpoint regions of CAMK2D (AP92/93), RNF38-MELK (AP94/95), AMY1B (AP96/97), DIPK1A (AP98/99), and RN7SKP123-MTF2 (AP100/101). The green highlighted sequence indicates cryptic heptamers, whereas the purple highlighted sequence indicates cryptic nonamers. The pink or olive green highlighted sequence indicates the sequence of cryptic RSS DNA substrates used in a gel-based assay. (B) Table represents the RIC (Recombination Information Content) score for the cryptic and canonical RSS sequences. (C) Native gel profile showing binding of purified RAGs with cryptic RSS DNA substrates derived from CAMK2D, RNF38-MELK, AMY1B, DIPK1A, and RN7SKP123-MTF2. “NC” indicates negative control substrates with no cryptic RSS. (D) The bar graph shows the quantification of RAG binding shown in panel B (*n* = 3). The error bar was calculated as mean ± SEM. ∗*p* < 0.05, ∗∗*p* < 0.005, ∗∗∗*p* < 0.0001, and “ns” is nonsignificant. (E) Denaturing gel profile shows cleavage by cRAGs on DNA substrates harboring cryptic RSS described in panels A and B. RAG nicking and the potential hairpin products are marked by gray and blue arrows, respectively. (F) The bar graph shows the quantification of the RAG cleavage product is shown in panel D (*n* = 3). The error bar was calculated as mean ± SEM. ∗*p* < 0.05, ∗∗*p* < 0.005, ∗∗∗*p* < 0.0001, and “ns” is nonsignificant. (G) Denaturing gel profile shows the impact of Mn^2+^ on RAG cleavage of DNA derived from GBM fragile regions containing cryptic RSS. DNA substrate derived from DIPK1A was incubated with cRAGs in the presence of an increasing concentration of MnCl_2_. DNA substrate AKN1/2 harboring standard 12RSS was used as the positive control (denoted as P). The nicked and the potential hairpin products are marked by gray and blue arrows, respectively. “M” is the Klenow ladder used as a 1 nt ladder. (H) Bar graph shows the quantification of RAG nicking product upon incubation with the increasing concentration of MnCl_2_ (*n* = 3). The error bar was calculated as mean ± SEM. ∗*p* < 0.05, ∗∗*p* < 0.005, ∗∗∗*p* < 0.0001, and “ns” is nonsignificant.

These breakpoint clusters were from genes CAMK2D (HGNC gene ID:1462, genomic location: hg38: chr4:113575580-113575639), intergenic region between RNF38 (HGNC gene ID:18052) and MELK (HGNC gene ID:16870) (genomic location: hg38: chr9:36519686-36519744; indicated as RNF38-MELK), Inc-AMY1B- 1 (genomic location: hg38: chr1:103489295-103489353; indicated as AMY1B), DIPK1A (HGNC gene ID:32213, genomic location: hg38: chr1:92913488-92913547), and intergenic region between RN7SKP123 (HGNC gene ID:45847) and MTF2 (HGNC gene ID:29535) (genomic location: hg38: chr1:93063051-93063098; indicated as RN7SKP123-MTF2) (Figures S3A–S3E). Importantly, most of the breakpoints observed were adjacent to cryptic 12 or 23RSS. Since cryptic signals were present near breakpoint regions, we hypothesized that RAGs may play a role in the generation of chromosomal rearrangements seen in these patients. Oligomeric DNA spanning cryptic RSS was designed from the selected patient fragile regions that were rich in breakpoints in such a way that the potential heptamer sequence was placed 21 nt away from the 5′ end. The oligomeric DNA was radiolabeled and annealed to their respective complementary sequence and used for RAG cleavage and binding studies. The double-stranded DNA substrates derived from different fragile regions were AP92/AP93 (CAMK2D; hg38: chr4:113575580-113575639), AP94/AP95 (RNF38-MELK; hg38: chr9:36519686-36519744), AP96/AP97 (AMY1B; hg38: chr1:103489295-103489353), AP98/AP99 (DIPK1A; hg38: chr1:92913488-92913547), and AP100/AP101 (RN7SKP123-MTF2; hg38: chr1:93063051-93063098) (Figure 4A). In the case of positive control, i.e., canonical RSS, the heptamer was 17 nt away from the 5′ end (Figure S3F). To evaluate the relative recombination potential of various cryptic recombination signal sequence (RSS) substrates, we calculated the RIC (Recombination Information Content) scores of the canonical and cryptic RSS harboring DNA substrates (Figure 4B). The canonical 12RSS sequence exhibited a RIC score of −15.75, which is within the range assigned typically for functional RSSs. In contrast, AP92/93, AP94/95, AP96/97, AP98/99, and AP100/101 substrates, which differ in spacer length and nucleotide composition, showed significantly lower RIC scores, ranging from −41.81 to −69.92. Notably, substrates with a 23 bp spacer (AP94/95, AP96/97, and AP98/99) exhibited more negative RIC values compared to their 12 bp spacer counterparts, indicating reduced RSS quality.

To investigate whether RAGs can bind to the fragile region using a biochemical assay system, GST-tagged core RAG1, and core RAG2 were co-expressed in the HEK293T cell line, and the protein was purified (Figures S4A and S4B), identity and activity were confirmed (Figures S4C–S4E).^32^^,^^50^ Purified cRAGs were incubated with radiolabeled DNA substrates covering fragile regions. An electrophoretic mobility shift assay (EMSA; 5% native PAGE) was performed to check the binding of RAGs following glutaraldehyde cross-linking of protein-bound DNA. Results showed that purified cRAGs could bind to all DNA substrates AP92/93, AP94/95, AP96/97, AP98/99, and AP100/101 containing cryptic RSS similar to the positive control, 12RSS; however efficacy of the binding was significantly higher in the case of canonical RSS (Figure 4C, left panel). Importantly, the RAG binding efficiency differed between fragile regions (Figure 4C), which was quantitated and presented (Figure 4D). The control DNA substrates DG13/14 and DG27/28, devoid of cryptic RSS, failed to show any RAG binding (Figure 4C, right panel).

The ability of the RAGs to cleave the fragile regions was assayed by incubating purified cRAGs along with 5′ end radiolabeled fragile regions from the breakpoint regions in a buffer containing 5 mM MgCl_2_ along with 1 mM MnCl_2_ (37°C for 1 h) and the products were resolved on 15% denaturing PAGE. Results showed distinct RAG nicking at substrates AP92/93, AP96/97, and AP98/99 at the expected position, i.e., at 21 nt away from the start site (Figure 4E). However, nicking efficiency varied among the substrates. It is important to note that the AP94/AP95 failed to show any nicking, although it had shown robust binding to RAGs. In contrast, the AP100/AP101 showed weak nicking (Figures 4E and 4F). Notably, substrates AP92/93 and AP98/99 exhibited potential hairpin product formation (Figure 4E, blue arrows), indicating RAG-mediated hairpin generation. Therefore, our results suggest that different fragile regions showed varying efficiency of the RAG cleavage, and this could be due to variations in the sequence of the cryptic signal.

Since the cleavage reaction was performed in the presence of 1 mM MnCl_2_, which was higher compared to the physiologic levels of Mn^2+^ (∼100 μM), the efficacy of cleavage in the presence of increasing concentrations of MnCl_2_ was assessed. The nicking assay was performed using substrate AP98/AP99 (DIPK1A) at different concentrations of MnCl_2_ (100, 200, 300, 400, and 500 μM). Results revealed that cleavage products were observed even at the lowest concentration of MnCl_2_ (100 μM), which is the physiological concentration of MnCl_2_ within cells (Figures 4G and 4H). Further, cleavage efficiency increased in a MnCl_2_ concentration-dependent manner (Figures 4G and 4H). Interestingly, increasing concentrations of MnCl_2_ led to a corresponding increase in the potential hairpin product formation, although this needs to be investigated further (Figure 4G).

### Fragile regions, when present on an extrachromosomal DNA, can undergo recombination within glioma cells

**Figure 5:**
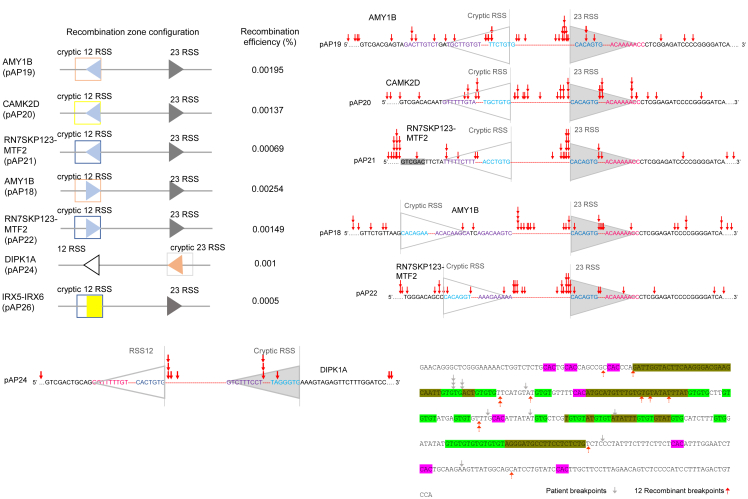
Episomal assay to evaluate the recombination potential of different fragile regions (A) The summary of the efficiency of recombination when different fragile regions associated with GBM were used in an extrachromosomal assay (*AMY1B*, *CAMK2D*, *RN7SKP123-MTF2*, *DIPK1A,* and *IRX5-IRX6*). (B) Cloning and sequencing of recombinant junction sequence of recombinants obtained after the transfection of recombination vectors pAP19 (cryptic RSS from *AMY1B* gene), pAP20 (*CAMK2D*), pAP21 (*RN7SKP123-MTF2*) in U87 cell line. (C) Sequence of the recombinant junction when vector pAP18 (cryptic RSS from *AMY1B* gene was cloned in reverse orientation), pAP22 (cryptic RSS from *AMY1B* gene was cloned in reverse orientation) was used for transfection into U87 cells. (D) Recombinant junction sequence of recombination vector pAP24 (DIPK1A). (E) Recombinant junction sequence of recombination vector pAP26 (IRX5-IRX6). Red arrows indicate the breakpoint obtained in recombinants and gray arrows indicate the breakpoints reported from the patients. CAC and GTG motifs are highlighted in pink and green, respectively. Cryptic RSS elements are highlighted in olive green color.

Based on the above results, we were interested in testing whether the fragile regions associated with GBM can undergo rearrangement in the glioma cells. To examine this, we cloned different fragile regions from GBM into pGG49 by replacing 12RSS, or 23RSS. Regions were cloned such that recombination between them and 12 or 23RSS will generate chloramphenicol resistance upon transformation into *E. coli* (Figure S5). Briefly, the fragile region AMY1B was cloned into pGG49 to generate the episome pAP19 (Figures 5A and S5A). This fragile region has 3 intrachromosomal structural variants of insertion, deletion, and inverted orientation type. The cloned region harbors a potential cryptic 12RSS with a heptamer and a nonamer with 3 nt mismatches each. Besides, there is a possibility of this region acting as a cryptic 23RSS, as an additional cryptic nonamer with 4 mismatches was seen after 23 nt from cryptic heptamer. The CAMK2D fragile region (hg38:chr4:113575580-113575639) was cloned into pGG49 vector to generate pAP20 (Figures 5A and S5B). This fragile region has 2 inter-chromosomal translocations and 1 intra-chromosomal translocation with an inverted orientation variant with a gap of 0.5 Mb. The partner gene involved in inter-chromosomal translocation here is Cadherin 11. The cloned region harbors a potential cryptic 12RSS consisting of a 3 nt mismatch heptamer and a 4 nt mismatch nonamer.

RN7SKP123-MTF2 fragile region was cloned into pGG49 to generate pAP21 (Figures 5A and S5C). The RN7SKP123-MTF2 fragile region has 4 structural variants of intrachromosomal insertion, deletion, and inverted orientation. The cloned region harbors cryptic 12RSS consisting of a 3 nt mismatch heptamer and 4 nt mismatch nonamer. The pAP24 was generated by cloning DIPK1A fragile region (hg38:chr1:92913488-92913547), which was in reverse orientation in pGG49 (Figures 5A and S5G). The DIPK1A fragile region has 2 intrachromosomal with inverted orientation type structural variants and harbors cryptic 23RSS consisting of 2 mismatch heptamer and 4 mismatch nonamer. The pAP26 construct has 368 bp breakpoint rich fragile region (11 breakpoint cluster) from intergenic region between IRX5 (HGNC gene ID:14361) and IRX6 gene (HGNC gene ID:14675) (hg38:chr16: 55034150–55034500; denoted as IRX5-IRX6) cloned to replace canonical 12RSS (Figures 5A and S5F). The pAP18 and pAP22 possess a cryptic RSS from AMY1B and RN7SKP123-MTF2 fragile regions, respectively, but both cloned in reverse orientation, whereas the pAP22 construct has a cryptic RSS cloned in reverse orientation (Figures 5A, S5D, and S5E).

In order to investigate the recombinogenic potential of the recombinants, the episomal constructs were transfected into U87 cells, and products were harvested after 48 h post-transfection using a Hirt-harvest method. Recombinants were identified following transformation into *E. coli* and by selecting on chloramphenicol-ampicillin plates (Figure S6A). Upon analyzing the data, we observed several double-resistant colonies, indicating successful recombination in U87 cells (Table S2). The recombination efficiency was calculated using the equation (CA/A∗100). The recombination efficiency of the AMY1B fragile region was 0.002% and 0.0025%, respectively, while for CAMK2D, it was 0.0014% (Figure 5A). The RN7SKP123-MTF2 fragile region showed a recombination frequency of 0.0007% and 0.001% for DIPK1A (Figure 5A). Intergenic fragile region between IRX5 and IRX6 region showed a recombination efficiency of 0.0005% when it was paired with a 23RSS. Overall, the recombination efficiency of the glioma fragile region was 200- to 500-fold lower than that of V(D)J recombination efficiency of B leukemic cell line, Nalm6, which was considered as a positive control upon analyzing on an extrachromosomal DNA substrate.

Further, CA-positive recombinants were analyzed using EcoRI restriction digestion for pAP19 (Figures S6A–S6D), pAP20 (Figures S6A, S6B, S6E, and S6F), pAP21 (Figures S6A, S6B, S6G, and S6H), pAP18 (Figures S6A, S6B, S6J, and S6K), pAP22 (Figures S7A and S7 B), pAP24 (Figures S7C and S7D), pAP26 (Figures S7E and S7F), followed by DNA sequencing. Results showed that most breakpoints were clustered adjacent to the heptamer though few were away from it, especially for fragile regions containing cryptic RSS (Figures 5B–5D). This may be attributed to RAG cleavage at heptamer followed by exonuclease action or DNA synthesis by error-prone DNA polymerase/s followed by ligation. For canonical RSS, breakpoint clustering was mostly observed before heptamer as per standard V(D)J recombination. In the case of pAP26 (368 bp breakpoint-rich; intergenic fragile region between IRX5 and IRX6 gene), almost all breakpoints were observed before heptamer (CAC/GTG) (Figure 5E). Future studies are required to understand the mechanisms governing the observed breakpoint clustering patterns, particularly when breakpoints occur distant from the heptamer sequence. It will be important to determine the role of different DNA double-strand break repair pathways, such as NHEJ and MMEJ, during the generation of recombinants.

### CRISPR/Cas9 mediated generation RAG1 deficient glioma cells abrogates the recombination of the fragile region

**Figure 6:**
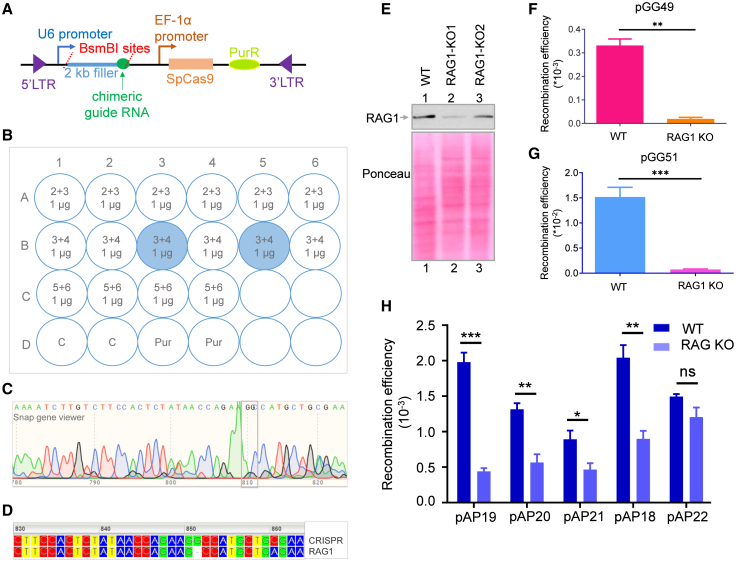
Generation of a knockout of RAG1 using CRISPR-Cas9 technology and evaluation of recombination efficiency upon abrogating RAG1 expression in the GBM cell line (A) Schematic of the lentiCRISPRv2 vector map indicating the sgRNA cloning site. (B) Representative wells show positive clones on a transfection plate. (C) Chromatogram following DNA sequencing shows a mutation at the RAG1 gene adjacent to the PAM site, confirming the generation of RAG1 knockout in the U87 cell line. (D) Blast sequencing result shows RAG1 ablated cells compared to the RAG1 WT sequence. (E) Western blot shows RAG1 expression in WT and RAG1 knockout cells. (F) Bar graph shows a reduction in recombination efficiency upon RAG1 KO for V(D)J vector (signal joint) pGG49 (*n* = 3). Error bar was calculated as mean ± SEM. (G) Bar graph shows a reduction in recombination efficiency upon RAG1 KO for V(D)J vector (coding joint) pGG51 (*n* = 3). The error bar was calculated as mean ± SEM. ∗*p* < 0.05, ∗∗*p* < 0.005, ∗∗∗*p* < 0.0001, “ns” non-significant. (H) Bar graph showing the reduction in recombination efficiency upon RAG1 KO (RAG1 ablated cells) for episomes harboring sequences from fragile regions (*n* = 3). Error bar was calculated as mean ± SEM.

Since the observed recombination described above did not precisely follow the V(D)J recombination pattern, we further investigated the role of RAGs in the recombination process. To do this, RAG1 KO cells were generated in U87 glioma cells using CRISPR-Cas9 technology. CRISPR technique introduces DNA double-strand breaks at the target locus, which then get repaired by either NHEJ machinery or HR. We designed guide RNA sequences spanning important regions such as GGTTTTCCGGATCGATGTGAAGG (just before NBD of RAG1, GTTCCGCTATGATTCAGCTTTGG (at the start of CD of RAG1), and TTGTGATGCCACCCGTCTGG at the ZnB of RAG1. Guide RNA sequences were designed and cloned into lentiCRISPRv2 (CRISPR-based lentiviral vector with puromycin selection marker and Cas9 gene) in the BsmBI restriction site (Figure 6A). The U87 cells were transfected with RAG1 sgRNA constructs followed by puromycin (0.5 μg/mL) selection for a month (Figure 6B). The clones that were puromycin resistant were propagated further and analyzed by western blotting for RAG1 expression, PCR, cloning, and sequencing to confirm the mutation in the RAG1 gene (Figures 6C–6E). The cell lysate was prepared from both puromycin-resistant U87 cells obtained, and U87 WT cells, and RAG1 expression was assessed by western blotting. Western blotting results showed that out of 2 puromycin-resistant U87 colonies obtained, RAG1-KO1 had no expression of RAG1 (Figure 6E). The same clone was checked by PCR and cloning, followed by DNA sequencing (Figures 6C and 6D). The DNA sequencing revealed a frameshift mutation that abrogated the reading frame of RAG1 (Figure 6D). Therefore, RAG1-KO1 cells in which RAG1 expression was ablated were used for recombination assay.

Extrachromosomal recombination assay was performed in RAG1 ablated U87 cells (referred to hereafter as U87-R1-1/3) and assessed for recombination efficiency for each recombination vector (Figures S5A–S5E). Experiments were performed independently (*n* = 3) with multiple transformations per batch. The cumulative graph was plotted for recombination constructs for pAP19, pAP20, pAP21, pAP18, and pAP22 (Figure 6H). We observed a significant reduction in the recombination frequency in the fragile regions, except for the pAP22 (Table S2A). The decrease was observed to be higher in the case of the pAP19 (cryptic RSS from AMY1B fragile region) vector (Table S2A). In the case of pAP24 (cryptic RSS from DIPK1A fragile region) and pAP26 (368 bp intergenic fragile region between IRX5 and IRX6 gene) constructs, basal recombination in WT U87 itself was at very low efficiency, and no recombination was detected in the case of RAG1 ablated cell line (Table S2C). In the case of pAP22, where the RN7SKP123-MTF2 fragile region was cloned in the reverse orientation, a few recombinant events were detected; however, these were not attributable to RAG-mediated recombination (Figure 6H). Analysis of sequencing results revealed the presence of only point mutations and small nucleotide deletions in these recombinants. Upon recombination assay using V(D)J plasmids pGG49 and pGG51, a significant reduction in recombination efficiency was seen in RAG1-KO cells as compared to the wild type (Figures 6F and 6G; Table S2B).

In summary, our results suggest that observed recombination is dependent on RAGs in glioblastoma cells. Further, this data indicates that RAGs present in glioblastoma cells are involved in generating chromosomal translocations, interstitial deletions, or rearrangements between fragile regions using a sequence-dependent recombination mechanism involving the RAG complex.

### Reconstitution of intrachromosomal rearrangement between AMY1B and RN7SKP123-MTF2

**Figure 7:**
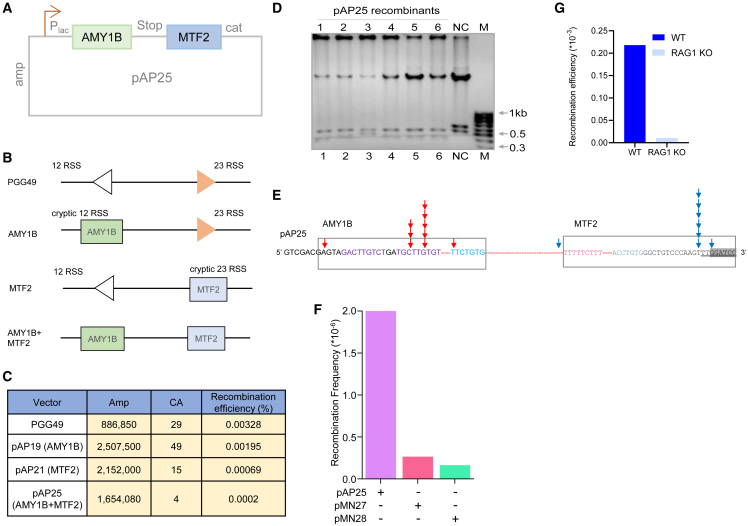
Evaluation of the recombination potential of AMY1B and RN7SKP123-MTF2 genes when they are present on an extrachromosomal DNA within cells (A) The schematic shows the extrachromosomal DNA substrate, pAP25, with AMY1B and RN7SKP123-MTF2 genes cloned into pGG49. (B) The schematic shows various episomal substrates used for the study, which include pGG49, pAP19, pAP21, and pAP25. (C) The table shows a summary of CA and Amp colonies obtained and recombination efficiency of only AMY1B and RN7SKP123-MTF2 fragile regions compared to when both the regions are present in the same episome, following the transfection of episomes in U87 cells. As a control, pGG49 was used for the study. (D) Agarose gel profile shows the restriction digestion pattern of the recombinants derived from pAP25. (E) Sequencing of recombinant junction derived from pAP25 showing the sequence of recombinants obtained after the transfection of pAP25. Red and blue arrows indicate the breakpoint obtained in recombinants at AMY1B and RN7SKP123-MTF2 genes. The rectangle indicates a portion of AMY1B and RN7SKP123-MTF2 fragile regions. (F) Bar graph depicts the recombination frequency in U87 cells following transfection with pAP25, pMN27, or pMN28 episomes. (G) Bar graph shows recombination efficiency between AMY1B and RN7SKP123-MTF2 genes when cells deficient in RAG1 were compared with their wild type U87 cells. In all the panels, RN7SKP123-MTF2 is indicated as MTF2.

Since chromosomal rearrangements generally occur between two genes within the same chromosomes or between two chromosomes, we were interested in mimicking the situation by cloning the genes of interest within the same episomes. The construct pAP25 was designed to mimic an intrachromosomal insertion-type structural rearrangement involving RN7SKP123-MTF2 and AMY1B, both from chromosome 1. The fragile regions, AMY1B and RN7SKP123-MTF2, were cloned to pGG49 by replacing the existing canonical 12RSS and 23 RSS, respectively (Figures 7A and S5H).

In order to evaluate the recombination potential of AMY1B and RN7SKP123-MTF2, an extrachromosomal recombination assay was performed in U87 cells by transfecting the episome pAP25. The episome harboring AMY1B (pAP19) or RN7SKP123-MTF2 (pAP21) fragile region alone served as the control (Figure 7B). pGG49 containing consensus 12 and 23 signal served as a positive control for V(D)J recombination. The recombination efficiency for the episomes was assessed as described above (Figure S6A). Results showed a recombination efficiency of 0.0002%, which was 10-fold and 3-fold lower than that of episome-bearing AMY1B (pAP19) and RN7SKP123-MTF2 (pAP21) fragile regions alone, respectively (Figure 7C). Further, the recombination frequency observed was >250-fold lower than that of pGG51, the positive control for V(D)J recombination (Figure 7C). Recombinants were further evaluated by restriction enzyme digestion followed by DNA sequencing (Figure 7D). Results revealed that while the breakpoints were seen at the coding-end region of the RN7SKP123-MTF2, it was at the signal end in the case of the AMY1B fragile region (Figure 7E). Interestingly, the majority of breakpoints were centered either at a particular nucleotide position or adjacent to it (Figure 7E). Despite the relatively low recombination frequency of the pAP25 episome, it was still around 8-fold and 13-fold higher than that of the control plasmids pMN27 and pMN28, respectively (Figure 7F). Further, the ablation of RAG1 significantly reduced recombination efficiency in pAP25, confirming the role of RAG1 (Figure 7G). In summary, we could reconstitute the intrachromosomal rearrangement between AMY1B and RN7SKP123-MTF2 and establish the role of RAGs in the generation of the deletion.

## Discussion

While chromosomal rearrangements are among the primary causes of glioblastoma, the precise mechanisms responsible for these genetic alterations remain largely unknown. The presence of cryptic RSS adjacent to the breakpoints in some of the genes that undergo such rearrangements indicated the potential of a V(D)J recombination-like mechanism, which made us question the involvement of RAGs in gliomagenesis. Few studies have proposed common fragile sites, palindromic duplication, transposons, and altered DNA repair as a cause behind these rearrangements.^11^^,^^51^^,^^52^^,^^53^^,^^54^^,^^55^ In addition, it was hypothesized that RAGs could be one of the players responsible for the same, as cryptic RSS was reported at the breakpoint region corresponding to the EGFRvIII deletion mutant, although they could not detect RAG expression.^56^ Cryptic RSS was seen at a breakpoint sequence of 1q32.1, which has been observed in a subset of glioblastoma.^47^ Thus, in the present study, we were keen to understand whether RAG is responsible for the genomic rearrangements observed in glioma.

In the present study, we have demonstrated the presence of RAG1 and RAG2 expression in multiple glioblastoma cell lines as well as glioblastoma patient samples. Our investigation provided compelling evidence affirming the expression of RAG1 and RAG2 in glioma cell lines, such as U251, U87, T98G, A172, U118, and LN229. The RNA-seq data analysis from 693 glioblastoma patient samples from the CGGA database showed that the vast majority of tumors expressed medium to high levels of RAG1 and RAG2, with no samples exhibiting simultaneous low expression of the two genes. This coordinated expression of RAG1 and RAG2 indicates that they share a common regulatory mechanism and possible co-dependency in glioma cells. This suggests that the components of the RAG complex are present in glioblastoma cells, raising the possibility of their involvement in genomic instability based on the previously reported association studies.^57^^,^^58^^,^^59^ Intriguingly, our immunofluorescence analysis revealed that RAG1 and RAG2 are predominantly localized within the nucleus of these cells, consistent with their anticipated role in DNA rearrangement.^38^^,^^60^ Importantly, we also observed that RAG1 present in the glioma cells could interact with RAG2, and it could catalyze a significant level of pathogenic recombination between conventional 12RSS and 23RSS within the glioblastoma cells, which was dependent on the activity of RAGs present in the cells.

By conducting an *in silico* analysis of the COSMIC database, we identified the existence of cryptic RSS within regions that undergo specific chromosomal rearrangements unique to brain cancer. Subsequent biochemical assays using purified RAGs demonstrated their ability to bind and cleave at cryptic RSS substrates derived from the fragile regions, such as AP92/93 (derived from the CAMK2D), AP94/95 (from the RNF38-MELK), AP96/97 (linked to the AMY1B), AP98/99 (associated with the DIPK1A), and AP100/101 (pertaining to the RN7SKP123-MTF2). The observations highlighted a direct link between the presence of RAGs and the potential cryptic RSS-mediated genomic rearrangements.

Notably, our extra-chromosomal recombination assay using fragile regions unveiled recombination events between these fragile regions and standard RSS, albeit at a much lower efficiency (100–500-fold lower) than that of standard V(D)J recombination (0.002% for AMY1B, 0.0013% for CAMKD2A, 0.0009% for RN7SKP123-MTF2). The sequencing of these recombinants confirmed that aberrant recombination events were occurring between fragile regions and RSS sequences. Most importantly, a reduction was observed in the recombination frequency upon knockout of RAG expression, indicating a direct link between RAGs and the occurrence of chromosomal deletions and rearrangements associated with glioblastoma. Overall, our findings strongly suggest the potential involvement of RAGs as one of the mechanisms underlying the genomic instability observed in glioblastoma, thereby providing valuable insights into the pathogenesis of this aggressive cancer.

The reconstitution of one of the rearrangements between AMY1B and RN7SKP123-MTF2 seen in glioma cells using an extrachromosomal translocation assay demonstrated that the recombination frequency between these genes was low compared to the frequency of either of the genes alone when paired with a consensus RSS. Furthermore, we observed that the recombination was dependent on the RAG complex during the gene rearrangement.

Consistent with our finding that cryptic RSS in the glioma fragile regions are the regions of RAG cleavage, previous studies have indicated the occurrence of breakpoints at or near cryptic heptamer or nonamer. For example, chromosomal rearrangements such as EGFRvIII deletion^56^^,^^61^ arise by the recombination of introns that contain sequences with homology to the RSS heptamers and nonamers present in the V(D)J region of the immunoglobulin and T lymphocyte antigen receptor genes. Another study reported the presence of cryptic RSS identified at the breakpoint sequence of 1q32.1, which subsequently underwent deletion, leading to glioma.^47^ Thus, our results are in line with the previous assumption that deletions mediated by RAG constitute a prominent mutational process characterized by the presence of recombination signal sequence motifs near breakpoints and the insertion of non-templated sequences at junctions.^62^ A comprehensive in-silico analysis of the COSMIC database further substantiated our findings by revealing the presence of cryptic RSS sequences in regions known to undergo chromosomal rearrangements specific to glioblastoma.

RAG proteins are key for initiating V(D)J recombination, a process in which double-strand breaks are finally generated precisely at the border between a recombination signal sequence (RSS) and a coding segment in B and T cells.^63^ The presence of the RAG complex in lymphatic cancer cells has been well-documented.^62^^,^^64^^,^^65^^,^^66^ Multiple studies have suggested the role of RAGs in the generation of chromosomal translocations associated with leukemia and lymphoma. Studies have shown that RAGs play a role in oncogenic transformation by mediating recombination between standard RSS of the Ig locus and cryptic RSS before the oncogene, leading to the overexpression of the oncogene under the Ig enhancer.^31^^,^^58^^,^^67^^,^^68^^,^^69^^,^^70^^,^^71^^,^^72^ In the (14;18) translocation resulting in follicular lymphoma, a study has shown that RAG mediated break at G4 DNA, the non-B DNA structure present in BCL2 MBR of chromosome 18.^58^^,^^73^ The studies have suggested a potential role for RAG1 in neurons, particularly in the site-specific recombination of elements within the neuronal genome^74^ or alternatively, preventing detrimental alterations of the genome in these long-lived cells. Besides, the expression of RAG1 has been reported in mouse embryonic brain tissues.^39^ This is consistent with the observed expression of RAGs in glioma cells, possibly due to the reactivation of it in tumorigenic conditions. Besides, it is also possible that glioma cells exploit this developmentally regulated machinery for adaptive genomic remodeling, facilitating tumor progression. The role of RAG1 and RAG2 in glioma cells, as well as the precise mechanisms underlying the regulation of RAGs in glioma, is currently being investigated in detail in our laboratory. However, further investigations are needed to dissect out the exact triggers for RAG activation in glioma and to delineate the downstream genomic consequences of such activity. Understanding these processes could uncover innovative therapeutics and help stratify patients with glioma based on RAG expression patterns or associated rearrangements. Therefore, targeting RAGs or their associated proteins/pathways may hold promise as a potential therapeutic strategy in managing deadly cancer and glioblastoma using specific drug delivery models.

In conclusion, our findings provide compelling evidence for the role of RAG1 and RAG2 in the generation of chromosomal deletions and rearrangements within glioma cells. Importantly, we established that both RAG1 and RAG2 are expressed in glioma cells, form a complex, and play a pivotal role in the generation of chromosomal deletions and rearrangements. These results not only enhance our understanding of the pathogenesis of this aggressive cancer but also underscore the potential relevance of the V(D)J recombination machinery in solid tumors, a concept hitherto primarily associated with glioblastoma.

### Limitations of the study

While our study provides strong evidence for the expression of RAG complex (RAG1/RAG2) in glioma cells and cleavage at cryptic RSS within fragile regions leading to chromosomal rearrangements, there are certain limitations. Although we have demonstrated the RAG-mediated recombination events using biochemical and extrachromosomal assays within glioma cells, the system used may not fully capture the complexity of chromatin context and nuclear architecture. We showed the coexpression and interaction of RAG1 and RAG2 in glioma cells; however, the upstream regulatory mechanisms leading to their reactivation in the non-lymphoid cells are yet to be elucidated. Additionally, our study does not rule out the involvement of other genomic instability factors or DNA repair defects that may cooperate with RAG activity. Finally, the direct contribution of RAGs in glioma genesis, if any, remains to be established.

## Resource availability

### Lead contact

Further information and requests for resources and reagents should be directed to and will be fulfilled by the lead contact, Sathees C. Raghavan (sathees@iisc.ac.in).

### Materials availability

All unique and stable materials generated in this study are available from the lead contact upon request and will be provided under a standard material transfer agreement.

### Data and code availability

All data relevant to the article are presented as main figures and supplementary figures. Any additional information related to the data reported in this article will be shared by the lead contact upon request. This article does not report original code.

## Acknowledgments

We thank Dr. Shivangi Sharma, Ms. Laijau Goyari, and members of the SCR laboratory for their critical reading and comments on the article. We also thank Ms. Divya Sathees, Mr. Annamalai Nataraj, and Mr. Aazim Hashmi for the technical help. This work was supported by a Center of Excellence Grant from the Department of Biotechnology (DBT), New Delhi, India (BT/PR13458/COE/34/33/2015) to SCR and BC; DBT (BT/PR52902/MED/30/2556/2024) to SCR; DBT (BT/PR40241/BTIS/137/70/2023) to BC. SCR is a JC Bose National Fellow (JCB/2023/000041). DST-FIST (SR/FST/LSII-045/2016) is also acknowledged for its support in maintaining the Central Facility of Biochemistry, IISc. AP and SK are supported by a Senior Research Fellowship (SRF) from IISc, India. LRS is supported by DST-Inspire, India fellowship.

## Author contributions

Conceptualization, SCR; design of experiments, SCR, AP,and SK; investigation, AP, SK, LRS, AM, SwK, ASM, NMN, and BC; writing, SCR, AP, SK, and AM; funding acquisition, SCR and BC; resources, SCR; supervision, SCR and BC.

## Declaration of interests

The authors declare that they have no conflicts of interest with the contents of this article.

## STAR★Methods

### Key resources table

**Table:** 

REAGENT or RESOURCE	SOURCE	IDENTIFIER
**Antibodies**		

RAG1	Santa Cruz Biotechnology	SC-363; RRID: AB_632307, SC-377127; RRID: AB_631727
RAG2	Santa Cruz Biotechnology	SC-5600; RRID: AB_2100414
GAPDH	BioLegend	631402; RRID: AB_2107422
GST	Santa Cruz Biotechnology	SC-138; RRID: AB_627677

**Critical commercial assays**		

Gel extraction kit	Qiagen	28704
TA Cloning kit	Qiagen	1113267
Plasmid midi extraction kit	Qiagen	12143

**Chemicals, peptides, and recombinant proteins**		

RPMI1640	MP Biomedicals	091260354
DMEM	MP Biomedicals	091233354
FBS	GIBCO	A52567-01
Penicillin Streptomycin	GIBCO	15140–122
Trypsin-EDTA	GIBCO	25200–072
Tetracyclin	Sigma	200-593-8
Puromycin	Sigma	200-387-8
Opti-MEM	GIBCO	31985070
DAPI	Sigma	D9542
Sephadex G-25	Sigma	S5772
NaCl	Sigma	S9625
γ-^32^P-ATP	BRIT	N/A
BamH1	NEB	R0136
Sal1	NEB	R0138S
T4 Polyneucleotide kinase	NEB	M0201S
EcoR1	NEB	R0101
Glutathione-agarose resin	Sigma	G4510
Glutathione	Sigma	G4251
MOPS	Sigma	M1254
DMSO	Sigma	D4540
DTT	Sigma	D9779
Potassium Glutamate	Sigma	G1501
Glycerol	Sigma	G5516
BSA	Sigma	A8806
Gluteraldehyde	Sigma	G5882
MgCl2	Sigma	M8266
MnCl2	Sigma	M3634
PEI	Sigma	764647
Potassium Acetate	Sigma	P1190
TRIS	Sigma	T1503
Leupeptin	Sigma	L9783
Pepstatin	Sigma	77170
Aprotinin	Sigma	A6279
PMSF	Sigma	P7626
Praformaldehyde	Sigma	158127
DABCO	Sigma	D27802
Trizol	Sigma	T9424
DNase	NEB	M0303
M-MuLV Reverse Transcriptase	NEB	M0253L
SYBR Green PCR mix	Takara	4344463
RNAse A	Sigma	10109142001
PIPES	Sigma	P6757
Glycogen	ThermoFisher	AM9510
Ampicilin	Sigma	A61040
Chloramphenicol	Amresco	0230
Kanamycin	Sigma	K1377
Proteinase K	SRL	49936

**sgRNA**		

AP9/AP10	This Paper	N/A
AP11/AP12	This Paper	N/A
AP13/AP14	This Paper	N/A

**shRNA construct**		

D10 (RAG1)	Resource Center, IISc, Bangalore	TRCN0000003433
E2 (RAG1)	Resource Center, IISc	TRCN0000003436
D3 (RAG2)	Resource Center, IISc	TRCN0000051231
D4 (RAG2)	Resource Center, IISc	TRCN0000051232
SHC002 (scrambled vector)	Resource Center, IISc	N/A
SHC001 (empty vector)	Resource Center, IISc	N/A

**Experimental models: Cell lines**		

U87 MG	National Center for Cell Science (NCCS), Pune, India	N/A
U-118 MG	ATCC	N/A
U251	IISc, Bangalore (KSS)	N/A
LN-229	ATCC	N/A
T98G	IISc, Bangalore (KSS)	N/A
SVgP12	ATCC	N/A
Nalm6	University of Southern California, (ML)	N/A
A172	IISc, Bangalore (KSS)	N/A
NHA	IISc, Bangalore (KSS)	N/A
REH	University of Southern California, (ML)	N/A
NHA	IISc, Bangalore (KSS)	N/A
HEK293T	NCCS	CRL-3216

**Bacterial and viral strain**		

*E.coli* DH5 α	N/A	N/A
*E.coli* DH10 β	N/A	N/A

**Oligonucleotides**		

AKN1, 5′-GATCAGCTGATAGCTACCACAGTGCTACA-GACTGGAACAAAAACCCTGCT-3′	Barcode Biosciences	N/A
AKN2, 5′-TAGCAGGGTTTTTGTTCCAGTCTGTAGCA-CTGTGGTAGCTATCAGCTGAT-3′	Barcode Biosciences	N/A
AP4, 5′-GGTTGGCAGGCCGGATATTA-3′	Barcode Biosciences	N/A
AP9, 5′-CACCGGTTTTCCGGATCGATGTGA-3′	Barcode Biosciences	N/A
AP10, 5′-AAACTCACATCGATCCGGAAAACC-3′	Barcode Biosciences	N/A
AP11, 5′-CACCGTTCCGCTATGATTCAGCTT-3′	Barcode Biosciences	N/A
AP12, 5′-AAACAAGCTGAATCATAGCGGAAC-3′	Barcode Biosciences	N/A
AP13, 5′-CACCGTCTTTGTGATGCCACCCGTC-3′	Barcode Biosciences	N/A
AP14, 5′-AAACGACGGGTGGCATCACAAAGAC-3′	Barcode Biosciences	N/A
AP19, 5′-GGCTTCTGGCTCAGTCTACA-3′	Barcode Biosciences	N/A
AP20, 5′-CTCAGCATGGCTTCTGGTTA-3′	Barcode Biosciences	N/A
AP21, 5′-CTGCCCTACTTGTGATGTGG-3′	Barcode Biosciences	N/A
AP22, 5′-GATGGATGAGTGTGCGTTCT-3′	Barcode Biosciences	N/A
AP32, 5′-CCGTGTCAACACCTTCCTCA-3′	Barcode Biosciences	N/A
AP33, 5′-TCCCATGCTTCTCACTCACG-3′	Barcode Biosciences	N/A
AP34, 5′-ACTGTGGTGGTGAAGGAGTC-3′	Barcode Biosciences	N/A
AP36, 5′-CCCTCTGCCAGTACAGTTTCA-3′	Barcode Biosciences	N/A
AP37, 5′-TCATTTCCCTCACTTGCCCA-3′	Barcode Biosciences	N/A
AP38, 5′-ACCTCCCTCCTCTTCGCTAC-3′	Barcode Biosciences	N/A
AP56, 5′-ACCTTAACCGCCTTATTAGCCA-3′	Barcode Biosciences	N/A
AP57, 5′-ACATTCAGGGCTCCATCAAATC-3′	Barcode Biosciences	N/A
AP92, 5′-TCGACAGGGTTTAAATGAAGCACAGCAAA-GAAATTAGAGCTACAAAAACATTGTG-3′	Barcode Biosciences	N/A
AP93, 5′-TCGACACAATGTTTTTGTAGCTCTAATTTC-TTTGCTGTGCTTCATTTAAACCCTG-3′	Barcode Biosciences	N/A
AP94, 5′-GATCCGCTGTGAGCTGAGATCACTCCATT-GCACTCCAGCTGGTGCAACAGAGCAAAACTCCATCG-3′	Barcode Biosciences	N/A
AP95, 5′-GATCCGATGGAGTTTTGCTCTGTTGCACC-AGCTGGAGTGCAATGGAGTGATCTCAGCTCACAGCG-3′	Barcode Biosciences	N/A
AP96, 5′-TCGACTAGTGTTCTGTTAAGCACAGAACAA-TTTTACAGAACACAAGCATCAGACAAGTCTACTCG-3′	Barcode Biosciences	N/A
AP97, 5′-TCGACGAGTAGACTTGTCTGATGCTTGTG-TTCTGTAAAATTGTTCTGTGCTTAACAGAACACTAG-3′	Barcode Biosciences	N/A
AP98, 5′-GATCCAAAGAACTCTACTTTCACCCTAATTA-ATACAAAAGGAGCCAATAATAGGAAAGACAAAATG-3′	Barcode Biosciences	N/A
AP99, 5′-GATCCATTTTGTCTTTCCTATTATTGGCTCC-TTTTGTATTAATTAGGGTGAAAGTAGAGTTCTTTG-3′	Barcode Biosciences	N/A
AP100, 5′-TCGACAAACTTGGGACAGCCCACAGGTA-GGAAGCAAGAGAAAGAAAAATAGAAG-3′	Barcode Biosciences	N/A
AP101, 5′-TCGACTTCTATTTTTCTTTCTCTTGC-TTCCTACCTGTGGGCTGTCCCAAGTTTG-3′	Barcode Biosciences	N/A
AP106, 5′-GCGCGTCGACGAACAGGGCTCGGG-AAAAAC-3′	Barcode Biosciences	N/A
AP107, 5′-GTACGTCGACTGGACAGTCTAAAG-GATGGGG-3′	Barcode Biosciences	N/A
DG13, 5′-GATCCCTCTAGACCGGTACTACTCGAGCC-ACACCCGCCCGCTGCACCCTCCTCCC-3′	Barcode Biosciences	N/A
DG14, 5′-GGGCGGGAGGAGGGTGCAGCGGGCGGG-TGTGGCTCGAGTAGTACCGGTCTAGAGG-3′	Barcode Biosciences	N/A
DG27, 5′-GATCCCTCTAGACCGGTACTACTCGA-GCGGCCCGGCGCTGCCAGCGCGGGCTCGG-3′	Barcode Biosciences	N/A
DG28, 5′-GGGCCCGAGCCCGCGCTGGCAGCGC-CGGGCCGCTCGAGTAGTACCGGTCTAGAGG-3′	Barcode Biosciences	N/A
KKC11, 5′-GCCTGTATCCAACACTTCG-3′	Barcode Biosciences	N/A
KKC12, 5′-AGCGTCGTGATTAGCGATG-3′	Barcode Biosciences	N/A
MS3, 5′-TTTTTTTTTTTTTTTTTTTTTTTTTTTTTTTTT-TT-3′	Barcode Biosciences	N/A
SS46, 5′-TCCATTGGAGGGCAAGT-3′	Barcode Biosciences	N/A
SS47, 5′-ACGAGCTTTTTAACTGCAGCAA-3′	Barcode Biosciences	N/A
SK19, 5′-CCCGCCATGATCTACTGCTC-3′	Barcode Biosciences	N/A
SK20, 5′-ACAGATGGATGAGTGTGCGT-3′	Barcode Biosciences	N/A
SK23, 5′-GTCCCACCTGGGAATTCGTT-3′	Barcode Biosciences	N/A
SK24, 5′-GGGATCTTCTCGTCGCCATC-3′	Barcode Biosciences	N/A
MS20, 5′-TTTTTTTTTTGACCATTGGCGATCTCA-GCGTACGGACGACTTCGGATGACTTTTTTTTTT-3′	Barcode Biosciences	N/A
MS21, 5′-GTCATCCGAAGTCGTCCGTACGCTG-AGATCGCCAATGGTC-3′	Barcode Biosciences	N/A

**Recombinant DNA**		

pNMN2	This Paper	N/A
pNMN3	This Paper	N/A
pNMN4	This Paper	N/A
pGG49	This Paper	N/A
pGG51	This Paper	N/A
pMN27	Nambiar et al., 2012	N/A
pMN28	Nambiar et al., 2012	N/A
pAP18	This Paper	N/A
pAP19	This Paper	N/A
pAP20	This Paper	N/A
pAP21	This Paper	N/A
pAP22	This Paper	N/A
pAP23	This Paper	N/A
pAP24	This Paper	N/A
pAP25	This Paper	N/A
pAP26	This Paper	N/A

**Software and algorithms**		

GraphPad Prism v5.0 and 7.0	GraphPad Software	N/A
Multi Gauge (V3.0) software	Fujifilm Medical Systems.	N/A
Zen Lite	Carl Zeiss’s ZEN microscopy software.	N/A
CFX Maestro analysis software	Bio-Rad	N/A
Olympus FS Software	Olympus, Japan	N/A

### Experimental model and subject details

#### Cell lines

Human glioblastoma multiforme (GBM) cell lines, LN229, U118, and SVGp12 (Human immortalized glial cells) were purchased from ATCC, USA. Human glioblastoma cell lines A172, U251, and T98G and normal human astrocyte (NHA) were from Dr. K. Somasundaram, Department of Microbiology and Cell Biology, IISc. U87 (Human glioblastoma grade IV cell lines) and 293T (human embryonic kidney epithelial cell line) were purchased from the National Center for Cell Science, Pune, India. Nalm6 and Reh cells were from Dr. M. R. Lieber (USA).

U87, U251, A172, T98G, LN229, U118, SVGp12, NHA and 293T were cultured in DMEM medium, whereas Nalm6 and Reh cells were cultured in RPMI1640 supplemented with 10% fetal bovine serum (FBS), 100 μg/mL Penicillin, and 100 μg/mL streptomycin and incubated at 37°C in a humidified atmosphere containing 5% CO_2_ as described before (Dahal et al., 2022b; Gopalakrishnan et al., 2024). RAG1 knockout cell line, U87-R1-1/3, was generated within the lab using CRISPR/Cas9 technology, as detailed below (Kumari et al., 2023a; Kumari et al., 2021). All cell lines were tested for mycoplasma contamination (Dahal et al., 2022b). Cell line authentication was performed at the source center (Dahal et al., 2022b).

### Method details

#### Purification of the oligomers and annealing

Oligomeric DNA was purified on 8–15% denaturing PAGE, whenever needed, as described before (Vartak et al., 2018). Oligomers were 5′-end labeled using T4 polynucleotide kinase and further purified using a Sephadex G-25 size exclusion column as described (Sharma et al., 2025). Radiolabeled duplex DNA was prepared by annealing [γ-P^32^] radiolabeled strand with 3-fold excess unlabeled complementary strand in the presence of 100 mM NaCl and 1 mM EDTA by heating in boiling water bath for 10 min followed by gradual cooling at room temperature (RT) (Dahal et al., 2022a).

A substrate containing the 12-RSS sequence was prepared by annealing γ-^32^P-labeled AKN1 oligomer with unlabeled AKN2 (Nambiar and Raghavan, 2012b; Paranjape et al., 2022). Control substrate DG13/14 was prepared by annealing the 5′ end labeled DG13 with DG14, whereas DG27/DG28 was prepared by annealing the 5′ end labeled DG27 with DG28. Double-stranded DNA substrates derived from fragile regions associated with glioma used were, AP92/AP93 from CAMK2D (by annealing 5′ end labeled AP92 with AP93), AP94/AP95 from RNF38-MELK (by annealing 5′ end labeled AP94 with AP95), AP96/AP97 from AMY1B (by annealing 5′ end labeled AP96 with AP97), AP98/AP99 from DIPK1A (by annealing 5′ end labeled AP98 with AP99), and AP100/AP101 from RN7SKP123-MTF2 (by annealing 5′ end labeled AP100 with AP101). The sequences of oligomers used in the study are provided (Table S1).

#### Plasmid constructs

Plasmids encoding GST-tagged human core RAG1and RAG2 as well as pGG49 and pGG51 were a kind gift from Dr. M.R. Lieber (USA). The episomal substrates harboring only the 12RSS (pMN27) or only 23RSS (pMN28) were generated by digesting the pGG51 by BamHI or SalI, respectively. The digested vectors were then purified and self-ligated (Nambiar and Raghavan, 2012a). pAP19 plasmid was generated by cloning AMY1B fragile region (genomic location-hg38:chr1:103489295-103489353) into SalI restriction site of pGG49 by replacing existing 12RSS (Figure S5). pAP20 plasmid was generated by cloning CAMK2D fragile region (genomic location-hg38:chr4:113575580-113575639) into SalI restriction site of pGG49 to replace the existing 12RSS (Figure S6). pAP21 plasmid was generated by cloning RN7SKP123-MTF2 fragile region (genomic location-hg38:chr1:93063051-93063098) into SalI restriction site of pGG49 to replace the existing 12RSS (Figure S5). pAP18 and pAP22 plasmids were generated by cloning AMY1B and RN7SKP123-MTF2 fragile regions, respectively into SalI site of pGG49 in opposite orientation (Figure S5). pAP24 plasmid was generated by cloning DIPK1A fragile region (hg38:chr1:92913488-92913547) into BamHI restriction site of pGG49 by replacing existing 12RSS in reverse orientation (Figure S5). pAP25 plasmid was generated by cloning RN7SKP123-MTF2 fragile region (hg38:chr1:93063000-93063140) into BamHI site of pAP19 replacing the existing 23RSS (Figure S5). pAP26 plasmid was constructed by cloning intergenic fragile region between IRX5 and IRX6 gene cloned to replace canonical 12RSS (Figure S5). 368 bp region from the IRX5-IRX6 gene (hg38:chr16: 55034150–55034500) was amplified from Reh genomic DNA using primers, AP106 and AP107. The PCR product was cloned in the SalI site of pGG49 by replacing canonical 12RSS. Gene symbols used are based on HGNC-approved nomenclature aligned with hg38 annotations.

#### RNAseq data

Transcriptomic data were obtained from the Chinese Glioma Genome Atlas (CGGA) database (http://www.cgga.org.cn), which includes RNA sequencing data and patient information across various glioma subtypes. Expression levels of RAG1 and RAG2 were extracted from normalized RNA-seq data (reported as RPKM). To reduce variability RPKM values were transformed using log_2_ (RPKM +1) and were used for further analysis. To categorize patients based on RAG1 and RAG2 expression, the 25th and 75th percentiles of the respective gene expression distributions were used as cutoffs (Low: <25th percentile, Medium: 25th–75th percentile, High: >75th percentile). Each patient was assigned to one of nine possible RAG1 and RAG2 expression subgroups (https://doi.org/10.1016/j.gpb.2020.10.005). These groupings were visualized using a two-dimensional scatterplot, where RAG1 expression is shown on the X axis and RAG2 on the Y axis. Color coding was used to denote the nine main subgroups, excluding those with very low counts.

#### Overexpression and purification of core RAGs

The mammalian expression constructs harboring core RAG1 (cRAG1, amino acids 384–1040), and core RAG2 (cRAG2, amino acids 1–383), each fused with N-terminal GST-tag were used for expression (Paranjape et al., 2022; Raghavan et al., 2005). In brief, HEK293T cells were co-transfected with equal amounts of cRAG1 and cRAG2 constructs by calcium phosphate method (Nishana and Raghavan, 2012; Raghavan et al., 2005). Cells were harvested 48 h post-transfection. Proteins were purified using glutathione-agarose resin (Sigma). Cells were resuspended in lysis buffer (25 mM Tris-HCl pH 8, 150 mM KCl, 0.5 mM EDTA, 10 M ZnCl_2_, 10% glycerol, 0.05% Triton X-100). The cell lysate was prepared by sonication in the same buffer in the presence of protease inhibitors. The supernatant was incubated with glutathione-agarose beads for binding overnight at 4°C. Beads were washed in the same buffer twice and eluted with 10 mM reduced glutathione. Purified fractions were resolved on 8% SDS-PAGE and were checked by silver nitrate staining. Identity was confirmed by western blotting. The activity was checked by site-specific nicking on standard radiolabeled 12RSS substrate (AKN1/2) (Naik et al., 2010).

#### Electrophoretic mobility shift assay (EMSA)

EMSA was carried out as described previously (Paranjape et al., 2022; Sharma et al., 2025). In brief, radiolabeled DNA substrates were incubated with appropriate cRAGs in a buffer containing 22.5 mM MOPS-KOH (pH 7.0), 20% DMSO, 2.2 mM DTT, 50 mM potassium glutamate, 2% (v/v) glycerol and BSA (100 ng/mL) for 1 h at 25°C following which crosslinking was performed using 0.1% glutaraldehyde at 37°C for 10 min. In the control, the RAG reaction buffer alone was used. The DNA-protein complexes were then resolved on 5 or 6% native polyacrylamide gels. The gels were dried, and bands were visualized by FLA9000 phosphorImager (Fuji, Japan). Each experiment described in the present study was done a minimum of three independent times with complete agreement.

#### RAG cleavage assay

RAG cleavage was performed as described earlier in a buffer containing 25 mM MOPS (pH 7.0), 30 mM KCl, 30 mM potassium glutamate, 5 mM MgCl_2,_ and 1 mM MnCl_2_ (Naik et al., 2010; Nambiar and Raghavan, 2012b; Paranjape et al., 2022). Radiolabeled substrates were incubated with cRAGs for 1 h at 37°C. Reactions were terminated by adding loading dye containing formamide, followed by heating for 10 min at 95°C and later electrophoresed on 15% denaturing polyacrylamide gels. To assess manganese ion requirement in RAG nicking, the assay was also performed at increasing concentrations of MnCl_2_ (100, 200, 300, 400, 500 μM). Each experiment described in the present study was done a minimum of three independent times with complete agreement.

#### Preparation of klenow ladder

5′ end labeled Poly T, 35 mer oligomer MS3 was incubated in buffer (10 mM Tris-HCl (pH 8), 10 mM MgCl_2_, 150 mM NaCl, 1 mM DTT) with Klenow polymerase for digestion at 37°C for 1 h (Sebastian and Raghavan, 2016). 0.3 volume of reaction was removed at 15 min, 30 min and 1 h time points and terminated by adding an equal amount of formamide-containing dye. All 3 fractions were merged, diluted, and loaded alongside 15% denaturing PAGE in order to correlate RAG cleavage products.

#### Plasmid isolation and purification

For purification of each plasmid, *Escherichia coli* was cultured in 200 mL of Luria broth (HiMedia, USA) for 18 h at 37°C, after transformation. Isolation of plasmid DNA was performed by standard alkaline lysis method (denaturing method). Plasmid was further purified using phenol: chloroform (1:1) and precipitated using Isopropanol, as described previously (Kumari et al., 2023b). The pellet was dissolved in TE buffer (pH 8.0).

#### Human cell transfection

U87 cells (∼1 × 10^6^) were seeded in 100 mm plates and incubated at 37°C with 5% CO_2_ till 70% confluency. 10 μg of plasmid was transfected using linear PEI polymer (Polyethylenimine) of molecular weight 25 kDa (Sigma, 1 mg/mL) in Opti-MEM. Briefly, the plasmid construct was mixed with linear PEI at the ratio 1:2 respectively, in Opti-MEM and incubated for 20 min at room temperature (Kumari et al., 2021). The resultant mixture was added dropwise to cells and incubated at 37°C with 5% CO_2_. Cells were harvested 48 h post-transfection.

#### CRISPR-Cas9 mediated generation of RAG1 knockout in U87 cell line

To study the impact of RAG1 knockout on extrachromosomal recombination assay inside the cells, we used the CRISPR-Cas9 method to generate RAG1 knockout (Kumari et al., 2021). sgRNAs transcribed from AP9/AP10, AP11/AP12, and AP13/14, which could target RAG1 at different domains such as NBD (Nonamer Binding Domain), CD (Catalytic Domain), and ZFB (Zinc Finger Domain) with minimal off-target effect, were cloned in pLentiCRISPRv2 backbone (generating pNMN2, pNMN3, and pNMN4). Without donor plasmid, DSBs generated by sgRNA will be repaired by NHEJ-causing indels. U87 cells (1∗10^5^) were seeded in 24 well plates and transfected with a combination of sgRNA. Transfection was performed using linear PEI:DNA in ratio of 2:1 and incubated at 37°C for 48 h. Cell culture medium (DMEM) was changed, and all wells were subjected to 0.5 μg/mL puromycin selection. The medium was changed every 4 days for 4 weeks. The transfection plate was monitored for colony formation over the period. At the end of 3^rd^ week, clear colonies were seen in 2 wells. These colonies were propagated further. Genomic DNA was isolated from obtained puromycin-resistant colonies and sequenced to confirm genomic alteration. The cell-free extract was prepared from WT U87 cells and puromycin-resistant cells and checked for RAG1 expression by western blotting using an anti-RAG1. sgRNAs used in the study are listed (Table S1).

#### Preparation of cell-free extracts

Cell-free extracts were prepared as described earlier with modifications (Baumann and West, 1998). Cells were washed in ice-cold phosphate-buffered saline (PBS) and resuspended in 2 volumes of hypotonic buffer (10 mM Tris-HCl (pH 8.0), 1 mM EDTA, 5 mM DTT and protease inhibitors), homogenized and incubated for 20 min at 4°C. After that, 0.5 volume of high salt buffer (50 mM Tris-HCl (pH 7.5), 1 M KCl, 2 mM EDTA, 2 mM DTT) was added, homogenized, and incubated on ice for 10–15 min. Extracts were centrifuged at 4°C for 3 h at 42000 rpm using a TLA-100 rotor (Beckman ultracentrifuge, Optima MAX-TL model). The supernatant was dialyzed overnight against dialysis buffer (20 mM Tris-HCl (pH 8.0), 0.1 M potassium acetate, 20% v/v glycerol, 0.5 mM EDTA, 1 mM DTT, 0.1 mM PMSF). Extracts were aliquoted and stored at −80°C until use.

#### RIPA method of extract preparation and immunoblotting

Cells were collected, washed in ice-cold phosphate-buffered saline (PBS) and resuspended in RIPA buffer (25 mM Tris-HCl pH 7.5, 150 mM NaCl, 1% NP-40, 1% Sodium deoxycholate, 0.1% SDS and 1 μg/mL each of aprotinin, leupeptin, pepstatin, 1 M DTT, 0.1% PMSF). Lysates were prepared by sonication at 36% amplitude for 2 min with 1 s on and 1 s off cycle pulse, followed by centrifugation at 14000 rpm for 10 min at 4°C; supernatant was collected and stored at −20°C. Protein concentration was determined by Bradford assay. The quantity of protein loaded on the gel was further equalized on SDS-PAGE.

For immunoblot analysis, 20–30 μg of protein was resolved on 8–10% SDS-PAGE (Dahal et al., 2018; Iyer et al., 2016; Sharma et al., 2015). Following electrophoresis, proteins were transferred to an activated PVDF membrane (Millipore, USA), blocked with 5% skimmed milk powder or 1% BSA. Proteins of interest were probed with appropriate primary antibodies against RAG1 (sc-363, sc-377127), RAG2 (sc-5600), GAPDH (Biolegend #631402, sc-137179), and GST (sc-138)appropriate secondary antibodies, as per standard protocol. The blots were developed using a chemiluminescent substrate (Immobilon Western; Millipore), and images were acquired using a chemiluminescence documentation system (LAS 3000; Fuji, Japan).

#### Immunofluorescence

Immunofluorescence was performed as described previously. Coverslips were UV-sterilized for 30 min before use. ∼50,000 cells (U87, T98G or RAG1 knock out cells) were seeded per coverslip in 6-well plate in the appropriate medium for each cell type and grown at 37°C in a CO_2_ incubator till they adhered (12 h). Cells were given two washes with 1X PBS, followed by fixation in 2% paraformaldehyde (15 min) and permeabilization with 0.1% Triton X-100 (5 min) at room temperature. Blocking was done in PBST containing 0.1% BSA, and 10% FBS for 1 h at 4°C, following which cells were incubated with appropriate primary antibody (8 h) at 4°C. Following this, the samples were incubated with the appropriate secondary antibody, and detection of the fluorescence signal by Strep-FITC incubation. The cells were counterstained with DAPI, mounted with DABCO (Sigma), and imaged under a confocal laser scanning microscope (Zeiss LSM 880 with 63X magnification). The images were processed using Zen Lite software.

#### Streptavidin beads pull-down assay

Canonical 12RSS oligomeric substrate biotinylated-AKN1/2 was prepared by annealing 5′ biotinylated AKN1 with AKN2 oligomer. Control oligomeric DNA substrate that does not contain a conserved heptamer and nonamer was prepared by annealing 5′ biotinylated MS20 with MS21 oligomer. 20 μL Streptavidin beads, 4 μg biotinylated 12RSS or the control DNA substrate, and 30 μg CFE from U87 or Nalm6 cell line in PBSI buffer (1X PBS, 10 mM NaCl, protease inhibitors, and 0.5 mM PMSF) were incubated for 2 h on end-to-end rotator at 4°C, followed by centrifugation (4°C, 1500 rpm). Following this, beads were washed 3 times with PBSI for 20 min at 4°C, followed by centrifugation (4°C, 1500 rpm). Bound proteins were eluted using Laemmli loading buffer and analyzed on 8% SDS-PAGE as well as western blotting.

#### Preparation of cDNA and RT-PCR

Total RNA was extracted using the Trizol method (Rio, 2010). Cells were homogenized into single-cell suspension with the help of a pestle at 4°C; chloroform was added, mixed vigorously, and kept at RT for 5 min. Centrifugation was done at 14000 rpm (4°C for 20 min), the supernatant was transferred to the new vial, and an equal volume of isopropanol was added, mixed, and centrifuged at 14000 rpm (4°C for 30 min). The pellet was washed using ethanol at 14000 rpm (4°C for 20 min). RNA pellet was dried and resuspended in DEPC treated, autoclaved, double distilled water. The quality of RNA was checked on agarose gel (1%) and quantitated using Nanodrop. 3–5 μg of RNA was used for cDNA synthesis. Following DNase treatment of the RNA sample (37°C for 5 min), cDNA was prepared using M-MuLV reverse transcriptase, random hexamer, oligo(dT), and dNTP, and incubated at 37°C for 1 h. A reaction without reverse transcriptase (-RT) was also done for each sample as a control. After cDNA preparation, genomic DNA contamination was checked by performing RT-PCR (Reverse Transcriptase PCR) for housekeeping genes/internal-control gene, 18S rRNA.

#### Real-time (quantitative PCR)

RNA was isolated from different glioblastoma cell lines (lines (U251, U87, T98G, U118, LN229), an immortalized astrocyte cell line (SVGp12) and a pre-B cell leukemia cell line (Nalm6). cDNA was prepared using MMLV Reverse Transcriptase (New England Biolabs, USA). The samples were equalized using *18S rRNA* as the internal control, and real-time PCR was performed using cDNA and 2X SYBR green PCR mix on a Bio-Rad CFX96 Touch Real-Time PCR machine as described previously (Kumari et al., 2021; Paranjape et al., 2022). Ct values for all the samples were determined using Bio-Rad CFX Maestro analysis software, and the relative expression of RAG1 and RAG2 genes was analyzed using the 2^−ΔΔCt^ method. 2^−ΔΔCt^ was plotted for RAG1 and RAG2 genes were using GraphPad Prism.

#### RNase protection assay

RNAse protection assay was performed by hybridizing total RNA with a radiolabeled probe followed by RNAse A treatment. For making the probe, 200 ng eluted PCR product (RAG1: AP34-AP20, 483 bp, and RAG2: AP38-AP4, 495 bp) along with random primer was heat denatured and incubated with Klenow pol and [α-^32^P] ATP along with dCTP, dGTP, dTTP at 37°C for 30 min; following which labeled probe was purified using a Sephadex G-25 size exclusion column as per manufactures direction. Total RNA (∼20 μg) isolated from U87 and Nalm6 cell line and radiolabeled ∼500 bp probe (10^6^ CPM) was denatured at 80°C for 10 min followed by hybridization overnight in hybridization buffer (40 mM PIPES(pH 6.4), 0.1 mM EDTA, 0.4 M NaCl) at 65°C. Following hybridization, the reaction was subjected to RNase A digestion in a buffer (contains 10 mM Tris-HCl (pH 7.4), 300 mM NaCl, 5 mM EDTA, 40 μg/mL RNase A) for 37°C (1 h). Samples were purified using phenol: chloroform (1:1) followed by ethanol preparation in the presence of glycogen. Resultant products were resuspended in 10 μL of nuclease-free water and resolved on 1% agarose gel. Gels were dried and exposed, and bands were visualized after Scanning using FLA9000 phosphorImager (Fuji, Japan).

#### Preparation of electrocompetent cells

*E. coli* DH5α/DH10β were streaked on Luria Bertani agar plate, followed by overnight incubation at 37°C. Single colony was picked and inoculated for primary culture in 3 mL LB medium (HiMedia) without antibiotics. As a control, inoculums were made in the presence of ampicillin, and kanamycin to check for contamination. After culture growth at 37°C (8 h), the culture was expanded to 20 mL and incubated overnight at 37°C, 180 rpm. The following day, LB medium (1 L) was inoculated using 20 mL of previously made culture along with 10 mL of 2 M glucose and kept for incubation at 37°C, 180 rpm. The culture was grown till O.D._600_ 0.35, following which bacterial cells were pelleted down at 4000 rpm (20 min at 4°C). Pellets were thoroughly washed twice with chilled autoclaved double-distilled water at 4000 rpm (20 min at 4°C). Further bacterial pellets were resuspended in 20 mL of 15% glycerol (autoclaved), centrifuged at 4000 rpm (20 min at 4°C); cells were resuspended in 1–2 mL 15% glycerol, aliquoted and stored at −80°C (Raghavan et al., 2001). Efficiency of electrocompetent cells were checked by transforming 10 pg of pUC18 in 30 μL of competent cells by electroporation, recovered in LB (1 h) at 37°C, plated in ampicillin containing LB agar plate, and incubated overnight at 37°C. Efficiency was calculated as follows: Transformation efficiency = No. of colonies in Amp plate/amount of DNA in μg.

#### Extrachromosomal recombination assay

GBM cell line U87 or U87-R1-1/3 was cultured and transfected with episomal V(D)J constructs pGG49 (signal joint), pGG51 (coding joint), pMN27 (harboring only 23RSS), pMN28 (harboring only 12RSS), or fragile region-containing constructs (pAP19, pAP20, pAP21, pAP18, pAP22, pAP24, pAP25 and pAP26) in the presence of linear PEI and incubated for 48 h at 37°C, 5% CO_2_ condition (Kumari et al., 2021; Raghavan et al., 2001). The plasmid DNA was extracted using a modified Hirt harvest protocol and transformed in *E. coli* DH10β (Hirt, 1967). The transformation mixture was plated on ampicillin (Amp) and chloramphenicol-ampicillin (CA) LB agar plates. Recombination between 12RSS and 23RSS or fragile region and standard RSS confers antibiotic resistance. Recombination efficiency calculated by the formula (CA/A∗100) gives the measure of recovered substrate that underwent V(D)J recombination. All the experiments were performed with a minimum of 3 independent repeats. Change in the recombination efficiency is represented as a bar graph. To confirm recombination, plasmid DNA was isolated from a double-resistant colony and analyzed using EcoRI restriction digestion. Positive recombinants were sequenced (Medauxin sequencing, India) and analyzed.

#### Episomal DNA isolation from mammalian cells

Mammalian cells were harvested after 48 h of transfection of pGG49 (signal joint), pGG51 (coding joint), pMN27, pMN28 or fragile region constructs (pAP19, pAP20, pAP21, pAP18, pAP22, pAP24, pAP25 and pAP26) and spun down at 1200 rpm, (10 min at 4°C). The pellet was washed twice with chilled PBS at 1200 rpm, (20 min at 4°C). The cell pellet was suspended in Hirt buffer (10 mM Tris-HCl pH 8, 10 mM EDTA), SDS (0.06%) and mixed gently until cells were lysed as described previously (Sharma et al., 2025). Further, 0.25 V 5M NaCl was added, mixed gently for 2 min, and incubated overnight at 4°C. The next day, the sample was spun down at 14000 rpm (30 min at 4°C), the supernatant was transferred to the new vial, and proteinase K (100 μg/mL) was added and incubated for 2 h at 55°C. DNA was purified with phenol:chloroform (1:1) and precipitated by chilled ethanol in glycogen. DNA pellets were resuspended in 20 μL autoclaved double-distilled water and used for electroporation.

#### Restriction digestion and sequencing of recombinants

The CA colonies were grown individually in 3 mL Luria broth for 14 h at 37°C. Plasmid DNA was isolated and was subjected to EcoRI restriction digestion for 2 h at 37°C. The digested products were resolved on 1.2% agarose gel to confirm recombinants. Negative recombinants were supposed to give 658 bp and 540 bp insert release. In contrast, positive recombinants were expected to be around 405 bp insert release in the case of pGG49 and fragile region plasmids 328 bp insert release in the case of pGG51. Clones of interest were sequenced and analyzed.

### Quantification and statistical analysis

Densitometry quantification of immunoblots and gels was done using Multi Gauge (V3.0) software. A rectangle covering the band of interest was selected, and intensity was quantified. A similar rectangle was then placed over other bands of interest in each lane, quantified, and added when required. An equal area from the same blot lane, where no specific band was present, was used as the background and subtracted for plotting the bar diagram. The details about the number of cells seeded has been mentioned in the methodology section.

Statistical analysis was performed using Student’s t test by comparing the experimental set with the control sample set using GraphPad Prism 5.0 or 7.0 (GraphPad Software). The statistical details of experiments are presented in the relevant figure legends. *p*-value of <0.05 was considered significant. All the data are represented as mean ± SEM. ∗*p* < 0.05, ∗∗*p* < 0.005, ∗∗∗*p* < 0.0001. “ns” is not significant; “AU” is Arbitrary unit and is measured as photo-stimulated luminescence unit (PSLU).

### Additional resources

No additional resources were generated in the study.
