# Supplementary material for: Recombination between fragile regions associated with chromosomal rearrangements in glioblastoma can be mediated by RAGs
Source: iScience. 2025 Oct 21;28(11):113815. doi: 10.1016/j.isci.2025.113815 (PMC12682277; doi:10.1016/j.isci.2025.113815)
Supplement: Document S1. Figures S1–S7 and Tables S1 and S2 [file mmc1.pdf]

iScience, Volume *28*

Supplemental information

Recombination between fragile regions associated with chromosomal rearrangements in glioblastoma can be mediated by RAGs

Amita Paranjape, Susmita Kumari, Lipsa Rani Sahu, Amrita Mondal, Swapna Kunhiraman, Arun Sharma M, Namrata M. Nilavar, Bibha Choudhary, and Sathees C. Raghavan

Supplementary figure legends

Table S1. List of oligomeric DNA used in the study.

Table S2. Table showing recombination efficiency when episomes harboring different fragile regions derived from GBM were transfected into U87 cells. A. pAP19 (AMY1B), pAP20 (CAMK2D), pAP21 (RN7SKP123-MTF2), pAP18 (AMY1B Reverse**)** and pAP22 (RN7SKP123-MTF2 Reverse) were used for recombination assay. A summary of total ampicillin (Amp) resistant and chloramphenicol-ampicillin (CA) resistant colonies obtained, and the recombination frequency for each episome was shown for both U87 WT and RAG1 ablated cell lines. For each vector, the experiment was repeated 3 times with multiple transformations. **B, C.** Recombination efficiency in U87 for pGG49 and pGG51 (B), pAP24 (DIPK1A), pAP25 (AMY1B and RN7SKP123-MTF2) and pAP26 (IRX5-IRX6) (C). Table shows a summary of CA and Amp colonies and recombination frequency following transfection of episomes in U87 WT and RAG1 ablated cell lines. In each case, the experiment was repeated at least 3 times with multiple transformations.

Figure S1. Evaluation of RAG1 and RAG2 expression profiles in glioma cell lines and RAG1 knock out U87 cells. A, B. Immunofluorescence analysis was performed to assess the intracellular localization of RAG1 and RAG2 in GBM cell lines U87 (A) and T98G (B), using anti-RAG1 and RAG2. **C, D.** Comparative analysis of RAG1 and RAG2 expression level in the wild type U87 cells (C) and RAG1 knock out U87 cells (D). In all the panels, DAPI was used to stain the nuclei, and FITC-conjugated secondary antibodies specific to RAG1 and RAG2 were employed for visualization.

Figure S2. Restriction analysis and junctional sequence characterization of recombinant clones obtained following transfection in the U87 cell line. A. Flowchart showing restriction digestion profile. **B-D.** Gel profile showing EcoRI restriction digestion of recombinants derived from pGG49 (B, C) and pGG51 (D). Upon digestion, positive recombinant is expected to release a 405 bp (for pGG49), or 326 bp (for pGG51) insert in addition to 540 bp release from the vector backbone. In contrast, a fragment of 658 bp is expected to be released in the case of an unrecombined vector, although size can vary depending on sequence alteration at the breakpoint region. **E.** Sequencing results displaying recombinant junctions derived from pGG51 episomal substrate following transfection in U87 cells. The open triangle denotes the 12 RSS, while the closed triangle indicates the 23 RSS. The top sequence corresponds to the parental plasmid, and the sequences below represent the recombinant clones. The recombination junction is highlighted with pink and green colour, while the 12-RSS heptamer is shown in sky blue and its nonamer in purple. For the 23RSS, the heptamer and nonamer is depicted in blue and pink colour, respectively.

Figure S3. Sequence of the breakpoint region of fragile regions associated with GBM patients used for the present study and schematic presentation of DNA substrates. A-E. Sequences of 5 selected fragile regions related to glioblastoma. Each breakpoint is represented by an arrowhead at the breakpoint site. The blue and yellow highlighted sequence indicates cryptic heptamer in both orientations. Grey highlighted sequence indicates cryptic nonamers. Light green color sequences indicate the DNA sequence of cryptic RSS substrates used in gel-based assay. **F.** Schematic showing cryptic RSS substrate used in the recombination assay. The bottom panel shows the canonical 12RSS substrate. In both panels, recombination sequences are indicated using the open triangle.

Figure S4. Purification of cRAG1 and cRAG2 and evaluation of RAG activity on 12RSS. **A.** Schematic showing experimental strategy used for overexpression and purification of cRAG1 (384-1040) and cRAG2 (1-383), each fused with N-terminal GST tags. HEK293T cells were co-transfected with cRAG1 and cRAG2 constructs, and proteins were purified using glutathione-agarose resin after cell lysis and sonication. **B.** Silver stained SDS-PAGE gel profile showing purity of cRAGs when fractions 1 and 3 were tested. “M” is a molecular weight marker. **C.** Western blot showing confirmation of the identity of purified cRAGs. The identity of the proteins was checked using anti-RAG1 and anti-RAG2. Fractions 1 and 3 were tested in the study. **D.** Sequence of 12RSS substrate used for activity assay. The substrate was 5’ end labelled using $[\gamma^{32}P]$ ATP. **E.** Evaluation of cleavage efficiency of purified GST-cRAGs on 12RSS substrate (AKN1/2). Radiolabeled DNA substrates were incubated with core RAG proteins (cRAGs) for 1 h at 37°C. Reactions were terminated and cleaved products were resolved on 15% denaturing polyacrylamide gels. PC is the positive control. The fractions that exhibited the most efficient DNA cleavage activity were used for further assays. RAG nicked (17 nt) and the potential hairpin products are marked by grey and blue arrows, respectively.

Figure S5. Schematics showing vector maps of recombination vectors. **A.** Vector map of pAP19. AMY1B fragile region was cloned into pGG49 by replacing 12RSS. **B.** Vector map of pAP20. CAMK2D fragile region was cloned into pGG49 by replacing 12RSS. **C.** Vector map of pAP21. RN7SKP123-MTF2 fragile region (indicated as MTF2) was cloned into pGG49 by replacing 12RSS. **D.** Vector map of pAP18. 12RSS in pGG49 was replaced by AMY1B fragile region (cloned in opposite orientation). **E.** Vector map of pAP22. 12RSS in pGG49 was replaced by RN7SKP123-MTF2 fragile region (cloned in opposite orientation; indicated as MTF2). **F.** Vector map of pAP26. IRX5-IRX6 fragile region was cloned. **G.** Vector map of pAP24. DIPK1A fragile region was cloned, replacing cryptic RSS. **H.** Schematic showing episomal construct, pAP25 generated using “DNA strider”. pAP25 was

generated by cloning RN7SKP123-MTF2 fragile region (indicated as MTF2) into BamHI site of pAP19 replacing the existing 23RSS.

Figure S6. Restriction digestion analysis of recombinants derived from pAP19, pAP20, pAP21 and pAP18. A. Schematic showing chromosomal translocation assay used for evaluating summary of recombination potential of fragile regions derived from GBM patients. **B.** Schematic showing restriction map of recombinant plasmid and the size of the restriction fragments upon EcoRI digestion as shown in the lower panel. **C, D.** Schematic of episomal construct, pAP19 (C) and agarose gel showing restriction digestion profile of recombinants derived from pAP19 (D). **E, F.** Schematic of episomal construct, pAP20 (E) and agarose gel showing restriction digestion profile of recombinants derived from pAP20 (F). **G, H.** Schematic of episomal construct, pAP21 (G) and agarose gel showing restriction digestion profile of recombinants derived from pAP21 (H). **I, J.** Schematic of episomal construct, pAP18 (I) and agarose gel showing restriction digestion profile of recombinants pAP18 (J) **K.** Schematics of symbols used to indicate cryptic/canonical RSS.

Figure S7. Restriction digestion analysis of recombinants derived from pAP22, pAP24, and pAP26. A, B. Schematic of episomal construct, pAP22 (A) and agarose gel showing restriction digestion profile of recombinants derived from pAP22 (B). **C, D.** Schematic of episomal construct, pAP24 (C) and agarose gel showing restriction digestion profile of recombinants derived from pAP24 (D). **E, F.** Schematic of episomal construct, pAP26 (E) and agarose gel showing restriction digestion profile of recombinants derived from pAP26 (F). **G.** Schematics of symbols used to indicate cryptic/canonical RSS.

Table S1. Oligomers used in the present study

```
Sequences of oligomers:
AKN1: 5’-GATCAGCTGATAGCTACCACAGTGCTACAGACTGGAACAAAAACCCTGCT-3’
AKN2: 5’-TAGCAGGGTTTTTGTTCCAGTCTGTAGCACTGTGGTAGCTATCAGCTGAT-3’
AP4:  5’-GGTTGGCAGGCCGGATATTA-3’
AP9:  5’-CACCGGTTTTCCGGATCGATGTGA -3’
AP10: 5’-AAACTCACATCGATCCGGAAAACC -3’
AP11: 5’-CACCGTTCCGCTATGATTCAGCTT-3’
AP12: 5’-AAACAAGCTGAATCATAGCGGAAC-3’
AP13: 5’-CACCGTCTTTGTGATGCCACCCGTC-3’
AP14: 5’-AAACGACGGGTGGCATCACAAAGAC-3’
AP19: 5’-GGCTTCTGGCTCAGTCTACA-3’
AP20: 5’-CTCAGCATGGCTTCTGGTTA-3’
AP21: 5’-CTGCCCTACTTGTGATGTGG-3’
AP22: 5’-GATGGATGAGTGTGCGTTCT-3’
AP32: 5’-CCGTGTCAACACCTTCCTCA-3’
AP33: 5’-TCCCATGCTTCTCACTCACG-3’
AP34: 5’-ACTGTGGTGGTGAAGGAGTC-3’
AP36: 5’-CCCTCTGCCAGTACAGTTTCA-3’
AP37: 5’-TCATTTCCCTCACTTGCCCA-3’
AP38: 5’-ACCTCCCTCCTCTTCGCTAC-3’
AP56: 5'-ACCTTAACCGCCTTATTAGCCA-3'
AP57: 5'-ACATTCAGGGCTCCATCAAATC-3’
AP92: 5’-TCGACAGGGTTTAAATGAAGCACAGCAAAGAAATTAGAGCTACAAAAACATTGTG-3’
AP93: 5’-TCGACACAATGTTTTTGTAGCTCTAATTTCTTTGCTGTGCTTCATTTAAACCCTG-3
AP94: 5’-GATCCGCTGTGAGCTGAGATCACTCCATTGCACTCCAGCTGGTGCAACAGAGCAAAACTCCATCG-3’
AP95: 5’-GATCCGATGGAGTTTTGCTCTGTTGCACCAGCTGGAGTGCAATGGAGTGATCTCAGCTCACAGCG-3’
AP96: 5’-TCGACTAGTGTTCTGTTAAGCACAGAACAATTTTACAGAACACAAGCATCAGACAAGTCTACTCG-3’
AP97: 5’-TCGACGAGTAGACTTGTCTGATGCTTGTGTTCTGTAAAATTGTTCTGTGCTTAACAGAACACTAG-3’
AP98: 5’-GATCCAAAGAACTCTACTTTCACCCTAATTAATACAAAAGGAGCCAATAATAGGAAAGACAAAATG-3’
AP99: 5’-GATCCATTTTGTCTTTCCTATTATTGGCTCCTTTTGTATTAATTAGGGTGAAAGTAGAGTTCTTTG-3’
AP100:5’-TCGACAAACTTGGGACAGCCCACAGGTAGGAAGCAAGAGAAAGAAAAATAGAAG-3’
AP101:5’-TCGACTTCTATTTTTCTTTCTCTTGCTTCCTACCTGTGGGCTGTCCCAAGTTTG-3’
AP106:5’-GCGCGTCGACGAACAGGGCTCGGGAAAAAC-3’
AP107:5’-GTACGTCGACTGGACAGTCTAAAGGATGGGG-3’
DG13: 5’-GATCCCTCTAGACCGGTACTACTCGAGCCACACCCGCCCGCTGCACCCTCCTCCC-3’
DG14: 5’-GGGCGGGAGGAGGGTGCAGCGGGCGGGTGTGGCTCGAGTAGTACCGGTCTAGAGG-3’
DG27: 5’-GATCCCTCTAGACCGGTACTACTCGAGCGGCCCGGCGCTGCCAGCGCGGGCTCGG-3’
DG28: 5’-GGGCCCGAGCCCGCGCTGGCAGCGCCGGGCCGCTCGAGTAGTACCGGTCTAGAGG-3’
KKC11:5’-GCCTGTATCCAACACTTCG-3’
KKC12:5’-AGCGTCGTGATTAGCGATG-3’
MS3:  5’-TTTTTTTTTTTTTTTTTTTTTTTTTTTTTTTTTTT-3’
SS46: 5'-TCCATTGGAGGGCAAGT-3'
SS47: 5'-ACGAGCTTTTTAACTGCAGCAA-3’
SK19: 5’-CCCGCCATGATCTACTGCTC-3’
SK20: 5’-ACAGATGGATGAGTGTGCGT-3’
SK23: 5’-GTCCCACCTGGGAATTCGTT-3’
SK24: 5’-GGGATCTTCTCGTCGCCATC-3’
MS20: 5’-TTTTTTTTTTGACCATTGGCGATCTCAGCGTACGGACGACTTCGGATGACTTTTTTTTTT-3’
MS21: 5’-GTCATCCGAAGTCGTCCGTACGCTGAGATCGCCAATGGTC-3'
```

A

Vector	RAG1 status	Transfection	Transformations	Amp	CA	Recombination efficiency	Total Amp	Total CA	Recombination efficiency
pAP19	WT	Batch 1	2	1750500	32	0.001828	4764500	94	0.001973
		Batch 2	2	1514000	34	0.002246			
		Batch 3	4	1500000	28	0.001867			
	ablated	Batch 1	6	916750	4	0.000436	3255500	14	0.00043
		Batch 2	2	956750	5	0.000523			
		Batch 3	1	1382000	5	0.000362			
pAP20	WT	Batch 1	4	1290750	16	0.00124	4031750	53	0.001315
		Batch 2	2	1346000	20	0.001486			
		Batch 3	3	1395000	17	0.001219			
	ablated	Batch 1	8	881750	7	0.000794	2876500	16	0.000556
		Batch 2	4	1007000	5	0.000497			
		Batch 3	3	987750	4	0.000405			
pAP21	WT	Batch 1	2	1651000	11	0.000666	4633000	41	0.000885
		Batch 2	6	1503000	14	0.000931			
		Batch 3	5	1479000	16	0.001082			
	ablated	Batch 1	4	1093750	7	0.00064	2936750	14	0.000477
		Batch 2	3	956500	4	0.000418			
		Batch 3	5	886500	3	0.000338			
pAP18	WT	Batch 1	2	846000	19	0.002246	2464000	50	0.002029
		Batch 2	3	887750	15	0.00169			
		Batch 3	6	730250	16	0.002191			
	ablated	Batch 1	4	1071000	12	0.00112	2853250	26	0.000911
		Batch 2	4	850250	7	0.000823			
		Batch 3	1	932000	7	0.000751			
pAP22	WT	Batch 1	5	1606000	25	0.001557	4749000	71	0.001495
		Batch 2	5	1609000	24	0.001492			
		Batch 3	3	1534000	22	0.001434			
	ablated	Batch 1	2	381750	5	0.00131	1067750	13	0.001218
		Batch 2	3	321250	3	0.000934			
		Batch 3	3	364750	5	0.001371			

B

Vector	RAG1 status	Transfection	Transfor-mations	Amp	CA	Recombination efficiency	Total Amp	Total CA	Recombination efficiency
pGG49	WT	Batch 1	3	739000	24	0.00325	2168410	73	0.00336
		Batch 2	8	591410	17	0.00287			
		Batch 3	4	838000	32	0.00382			
	ablated	Batch 1	4	1569250	5	0.00032	4614000	9	0.00019
		Batch 2	3	1367750	2	0.00015			
		Batch 3	2	1677000	2	0.00012			
pGG51	WT	Batch 1	7	744600	140	0.01880	2522600	375	0.01486
		Batch 2	3	999600	122	0.01220			
		Batch 3	5	778400	113	0.01452			
	ablated	Batch 1	3	422750	3	0.00071	1584000	12	0.00076
		Batch 2	4	637000	6	0.00094			
		Batch 3	2	524250	3	0.00057			

C

Vector	RAG1 status	Transfection	Transformations	Total Amp	Total CA	Recombination efficiency
pAP24	WT	7	13	1288650	6	0.000466
	ablated	11	30	3097650	0	0
pAP25	WT	5	20	5513600	12	0.000218
	ablated	6	15	1846788	0	0
pAP26	WT	5	39	3690000	15	0.00041
	ablated	6	40	4094800	0	0

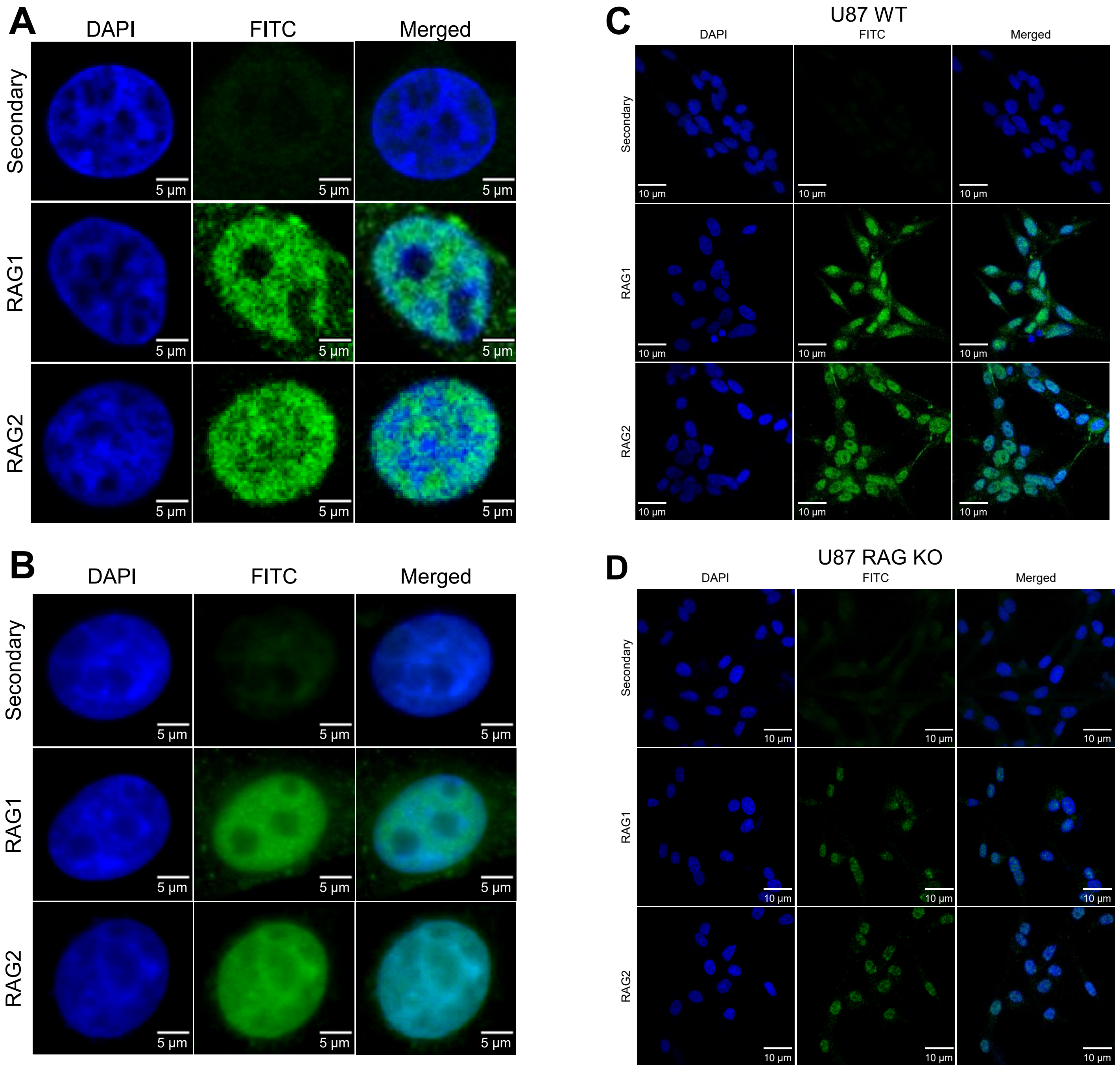
A
DAPI
FITC
Merged
Secondary
RAG1
RAG2
5 μm
B
DAPI
FITC
Merged
Secondary
RAG1
RAG2
5 μm
C
U87 WT
DAPI
FITC
Merged
Secondary
RAG1
RAG2
10 μm
D
U87 RAG KO
DAPI
FITC
Merged
Secondary
RAG1
RAG2
10 μm

Figure S1

Figure S2

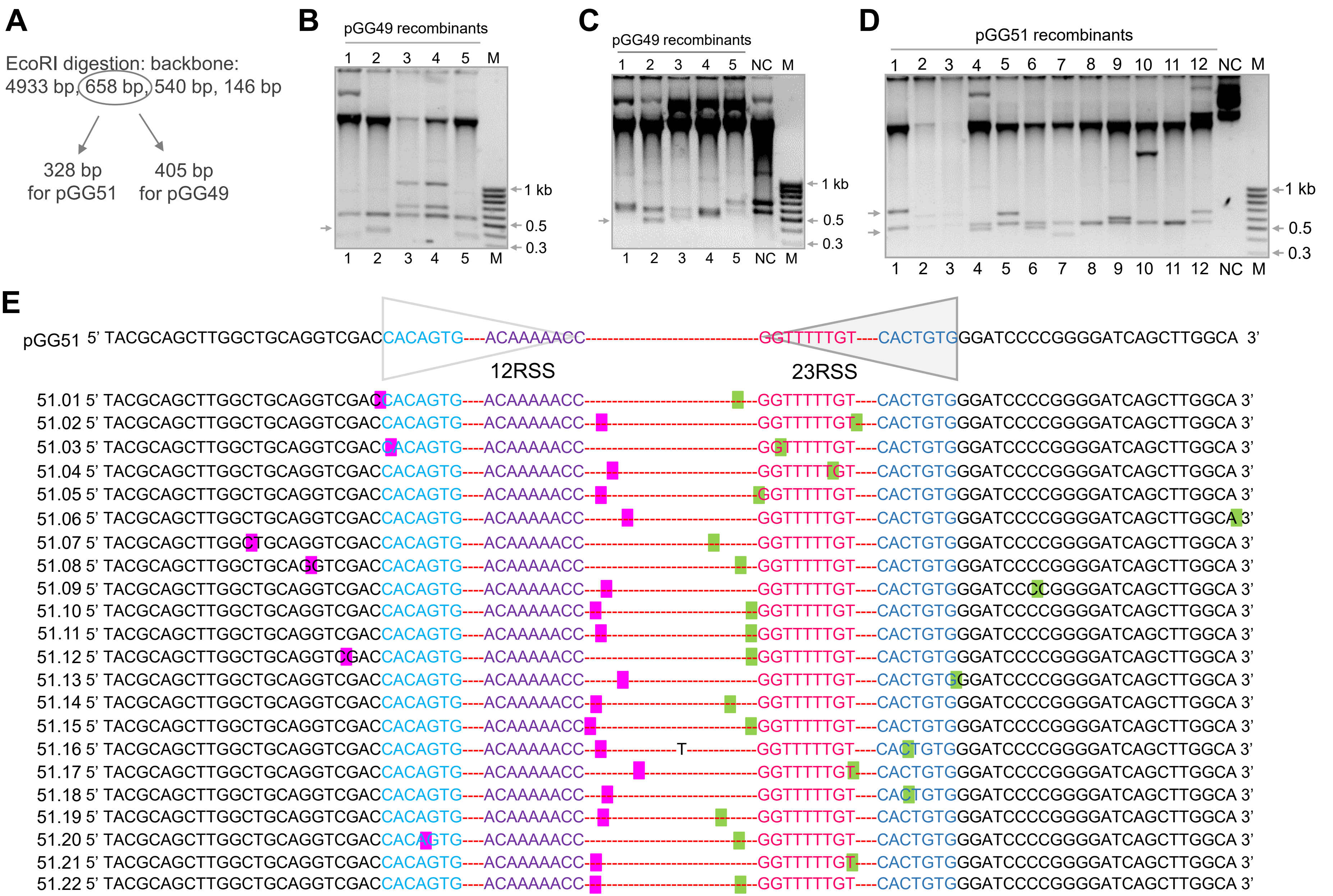

A
EcoRI digestion: backbone:
4933 bp, 658 bp, 540 bp, 146 bp
328 bp for pGG51
405 bp for pGG49
B
pGG49 recombinants
1 2 3 4 5 M
1 kb
0.5
0.3
C
pGG49 recombinants
1 2 3 4 5 NC M
1 kb
0.5
0.3
D
pGG51 recombinants
1 2 3 4 5 6 7 8 9 10 11 12 NC M
1 kb
0.5
0.3
E
pGG51 5' TACGCAGCTTGGCTGCAGGTCGACCACAGTG----ACAAAAACC----GGTTTTTGT----CACTGTGGGATCCCCGGGGATCAGCTTGGCA 3'
12RSS
23RSS
51.01 5' TACGCAGCTTGGCTGCAGGTCGACCACAGTG----ACAAAAACC----GGTTTTTGT----CACTGTGGGATCCCCGGGGATCAGCTTGGCA 3'
51.02 5' TACGCAGCTTGGCTGCAGGTCGACCACAGTG----ACAAAAACC----GGTTTTTGT----CACTGTGGGATCCCCGGGGATCAGCTTGGCA 3'
51.03 5' TACGCAGCTTGGCTGCAGGTCGACCACAGTG----ACAAAAACC----GGTTTTTGT----CACTGTGGGATCCCCGGGGATCAGCTTGGCA 3'
51.04 5' TACGCAGCTTGGCTGCAGGTCGACCACAGTG----ACAAAAACC----GGTTTTTGT----CACTGTGGGATCCCCGGGGATCAGCTTGGCA 3'
51.05 5' TACGCAGCTTGGCTGCAGGTCGACCACAGTG----ACAAAAACC----GGTTTTTGT----CACTGTGGGATCCCCGGGGATCAGCTTGGCA 3'
51.06 5' TACGCAGCTTGGCTGCAGGTCGACCACAGTG----ACAAAAACC----GGTTTTTGT----CACTGTGGGATCCCCGGGGATCAGCTTGGCA 3'
51.07 5' TACGCAGCTTGGCTGCAGGTCGACCACAGTG----ACAAAAACC----GGTTTTTGT----CACTGTGGGATCCCCGGGGATCAGCTTGGCA 3'
51.08 5' TACGCAGCTTGGCTGCAGGTCGACCACAGTG----ACAAAAACC----GGTTTTTGT----CACTGTGGGATCCCCGGGGATCAGCTTGGCA 3'
51.09 5' TACGCAGCTTGGCTGCAGGTCGACCACAGTG----ACAAAAACC----GGTTTTTGT----CACTGTGGGATCCCCGGGGATCAGCTTGGCA 3'
51.10 5' TACGCAGCTTGGCTGCAGGTCGACCACAGTG----ACAAAAACC----GGTTTTTGT----CACTGTGGGATCCCCGGGGATCAGCTTGGCA 3'
51.11 5' TACGCAGCTTGGCTGCAGGTCGACCACAGTG----ACAAAAACC----GGTTTTTGT----CACTGTGGGATCCCCGGGGATCAGCTTGGCA 3'
51.12 5' TACGCAGCTTGGCTGCAGGTCGACCACAGTG----ACAAAAACC----GGTTTTTGT----CACTGTGGGATCCCCGGGGATCAGCTTGGCA 3'
51.13 5' TACGCAGCTTGGCTGCAGGTCGACCACAGTG----ACAAAAACC----GGTTTTTGT----CACTGTGGGATCCCCGGGGATCAGCTTGGCA 3'
51.14 5' TACGCAGCTTGGCTGCAGGTCGACCACAGTG----ACAAAAACC----GGTTTTTGT----CACTGTGGGATCCCCGGGGATCAGCTTGGCA 3'
51.15 5' TACGCAGCTTGGCTGCAGGTCGACCACAGTG----ACAAAAACC----GGTTTTTGT----CACTGTGGGATCCCCGGGGATCAGCTTGGCA 3'
51.16 5' TACGCAGCTTGGCTGCAGGTCGACCACAGTG----ACAAAAACC----T----GGTTTTTGT----CACTGTGGGATCCCCGGGGATCAGCTTGGCA 3'
51.17 5' TACGCAGCTTGGCTGCAGGTCGACCACAGTG----ACAAAAACC----GGTTTTTGT----CACTGTGGGATCCCCGGGGATCAGCTTGGCA 3'
51.18 5' TACGCAGCTTGGCTGCAGGTCGACCACAGTG----ACAAAAACC----GGTTTTTGT----CACTGTGGGATCCCCGGGGATCAGCTTGGCA 3'
51.19 5' TACGCAGCTTGGCTGCAGGTCGACCACAGTG----ACAAAAACC----GGTTTTTGT----CACTGTGGGATCCCCGGGGATCAGCTTGGCA 3'
51.20 5' TACGCAGCTTGGCTGCAGGTCGACCACAGTG----ACAAAAACC----GGTTTTTGT----CACTGTGGGATCCCCGGGGATCAGCTTGGCA 3'
51.21 5' TACGCAGCTTGGCTGCAGGTCGACCACAGTG----ACAAAAACC----GGTTTTTGT----CACTGTGGGATCCCCGGGGATCAGCTTGGCA 3'
51.22 5' TACGCAGCTTGGCTGCAGGTCGACCACAGTG----ACAAAAACC----GGTTTTTGT----CACTGTGGGATCCCCGGGGATCAGCTTGGCA 3'

Figure S3

A

hg38:chr4:113575580-113575639: CAMK2D (AP92/93)

```
TTATTCTACTCATTTGCTGTCTTAAGGAACTTAAAAAGAAGGGTAATTATTTATTTGAAGATTA
CCATCCACTCCATGG▼(2)ATAGAAAGACAATAGAAGAA▼CTAATATTATG▼TGTCTGTATGA
TAAATTGCAGTGTTTTATGCAACAAAATGTAAAACAATTAA▼AGAAACACCTCATTCACTTGGT
ATCATTAGGGACAGGGCTATTTTCTTAAATTATTTTTCAAAATAACAAAAGCTCCTTTGAGCTC
CAAAACTTTATTTAATTAATTTTGATGGCTACAACAAAATATACTTTAAATTTCTCTGAATTCT
TAAATATGCAAAATATAATTTTCTTAAAGACCCCGGATCATAATTATGATTATACAATTCTG/T
GTTATGCAGTTTTTGGTGCTTAAGAACTACAGAGGTTAGTAGAAATATTTGGCCCCTCACCAAG
TCCTGTCAAA▼CCACAATGTTTTTGTAGCTCTAATTTCTTTGCTGTGCTTCATTTAAA▼(2)CC
CTCAGTCAGTCAGCTCTTTATTCTTCTTGTTATTGCAATCAGTGTATCTATGTCTTTGTCTTT
```

B

hg38:chr9:36519686-36519744: RNF38-MELK (AP94/95)

```
AP94/95ACCAGCTACTCGGGAGGCTGAGGCAGGAGATCACTTGAGCCTGGGAAGCAGAGGTTG
CTGTGAGCTGAGAT▼CAC▼TCCATTGCACTCCAGCTGGTGCAACAGAGCAAAACTCCATCTCA
AAACACACACACACACACACACACACACACACACACACA▼CAAAACCTATGTAGTCCACCCCGT
CCTTATTTCTGTTATCCTTTTTTTCCCTCAAGGACCAAGTGGCTTAAGCTTTTCTGACCAAATA
ACATTAATTTTATCTTTTATCCTCTCCCCATCCCTCTTCCCCTC▼CCCCTCCCCCTCTCTCTCC
CTCTCCCCCTC▼TTCTTCTTTCTTCTTCTTTTGAGACAGGGTCTTGCTCTGTTCCCCAGGCCAG
AGTACAGTGGCAGGATCTTTGCTCACTGCAACCCCCGCCTCCTGGGTTCAAATGATTCTCATGC
CTTAGCCTCCTGAGTAGCTAGGACTATAGGTATGCACCACCACACCTGGCTAATTTTTTGTGTT
TTTAGTAGAGACGGTTTCTCCATGTTGGAGGGGCT▼GCTCTTGTGACCTCAGGTGATCTGCCAG
CCTCAGGCTCCCAAAGTGCTGGGATTACAGGCATGAGCT
```

C

hg38:chr1:103489295-103489353: AMY1B (AP96/97)

```
TGGGTATCTCAAAAAATCTAATCTATAGAAAAGGCAGAGTAAAGTGAGGCTATGGCTATAATTG
TTAAAGAAGCAACAGTCAGCCGTATAGCTAGGAGAGGGAAGTGTTTGCTGACTTG▼CTTCTACG
TTGTTCATGTTTTGTGTTTAGACATGATTACAAAGTGGAACTCTTTTTGTTTTACTCCAT▼AAT
GGTCACAGAGTAGACTTGTCTGATGCTTGTGTTCTGTAAAATTGTTCTGTG▼CTTAACAGAACA
CTAAGACTTAA▼CTGTGAATGTGGGGCCAGTGCGTAGAAACACCAAAGCCTGGTTGATAGTACC
GGGTCAGCTCCC▼AGATGTCAAGGGCTAATTTTCCTTTCTTAGTATTGACTTATGAAATCAA▼C
TAATTGATTTTAATTGAGTAAACATATACATAGTGGCTTTGTTTTTACTGAATTTTATACTATA
TCTTCACTGCTATTGACACAGATGTGCTACTTAAT
```

D

hg38:chr1:92913488-92913547: DIPK1A (AP98/99)

```
GAAAAAGAGAGAA▼AAAAAAAGATAACAGAGTTAATCAGGCCATTATTTACAGCTATCTTATTC
ATGCCAAAACATGTTAGCTTCCTTTTTTAAAATC▼AAGTCACCAAGATGAGGTGTTAGCTTTCT
AATTGCAGGA▼TAAAATTCAATATTTAATGACTACAATAC▼TGCAGTTTACACAATTTAT▼T
CCCAAGCTACTGATTGCTTCAACCCCAGGTCAGTCACCTAAGTTGACAGCAGCCCTGTATGTTT
GCTCTAGTACTTTACAAATTA▼TCATTCTTT▼TATACCATGATGTGAAAAAAAAAAGGGGAAA
ATACTGGTCTATTCATTTGTGTACCT▼AGTTGGAGAACATTTCTGCTTCGAGAAGACAGATTCT
CAGTCAATAAATAAAAGATGGCAAAAATATCCATTTTGTCTTTCCTATTATTGGCTCCTTTTGT
ATTAATTAGGGTGAAAGT▼AGAGTTCTTTGCATGTTCCATGACACATTCTTTATAAGCAAATTA
AATAATCTCTAAAGTTCCTTCTTGCTCCATGAGCTGTAACTGAATTATTTTATTGAACTGACAC
ACAGAGGGCAGCAG▼TAAGTAATATACTGTATATGATACGTTATGTGCCTGGGGGCCTTAGCCA
ACTTCTCTGCCGCATAGCTCTCCCTCTAGCAAAGCTTTTTATTTTGGCCCTCTCTGCCTGTCTC
CTCTGATCTGTATCTTTCTTTCTCCTCCT▼GCCAGAGCTTTCTTCCCATTTTGTCAGACTTAGA
AAATCAAGAATAGATCAATATTACCATTCTGGAAAAAGGTAAGCTA
```

E

hg38:chr1:93063051-93063098: RN7SKP123-MTF2 (AP100/101)

```
GGGACTAAGGTGCACGCCACTGCACCTGGCTCCTCAGTTACATTTATTGGCTTCTATTTTTCTT
TCTCTTGCTTCCTACCTGTGG▼GCTGTCCCAAGTT▼TCAGTCCAT▼C▼ACTTGCTGTACTCT
AGGGACTTTGTTTCATGGAAA▼GTTCTTCTATTCTCACAATT▼TAAAAAC▼ATTTATGTTAA
CTATTTCTAAATAAACTTCTTTA▼TTCTCA▼CACTTACTTAAACTCCAAGCTTTTCCTCCCAA
TTCTATTTGAAATAGTTCTAATTG▼GGACGGTTC▼TAATCTCACTCTATCTCAAATTCAAAAC
GTCAAAAATAAGAGTAGTATCCCCAAAACAAAGTCATCATCTCCTTCTGAAAACTAGCTTCTC▼
CCAAGCTCCCTCTTTTTCTATATAATTATTCTTATTATTCCAGGCTTAGA▼ACTTTGGTGTCAT
CTTAAATTTCCCCCTTCTTTACTATAAACTATGTTTATAGATCACTTTCTGTTAT▼AGTT▼TC
TCTCTTTCAAAATTTCTATTAGATCCAGGCC
```

F

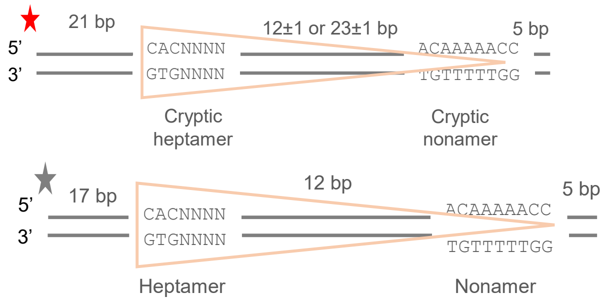

Figure S4

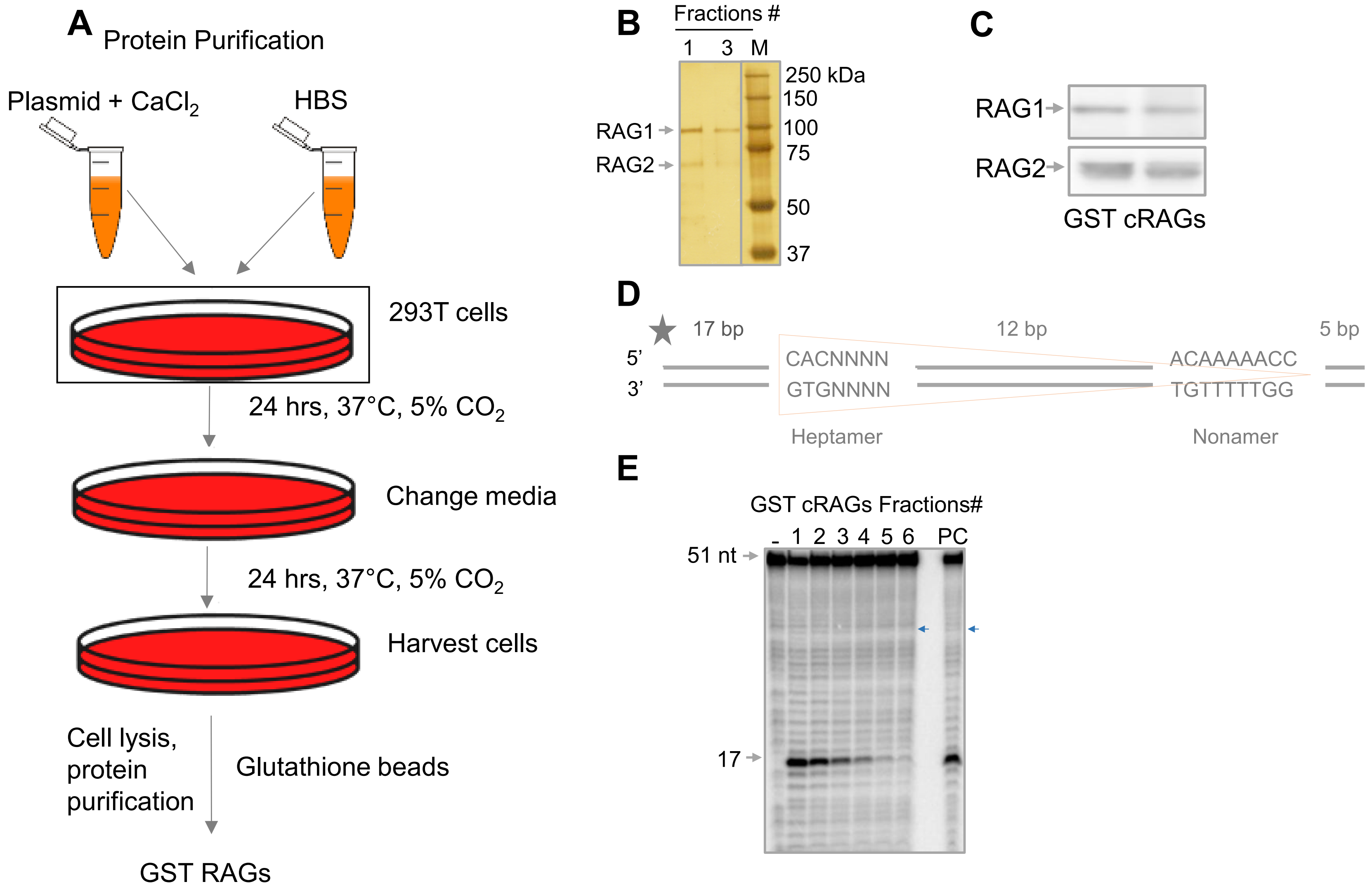

A
Protein Purification
Plasmid + CaCl2
HBS
293T cells
24 hrs, 37°C, 5% CO2
Change media
24 hrs, 37°C, 5% CO2
Harvest cells
Cell lysis, protein purification
Glutathione beads
GST RAGs
B
Fractions #
1 3 M
250 kDa
150
100
75
50
37
RAG1
RAG2
C
RAG1
RAG2
GST cRAGs
D
17 bp
12 bp
5 bp
5'
3'
CACNNNN
GTGNNNN
ACAAAAACC
TGTTTTTGG
Heptamer
Nonamer
E
GST cRAGs Fractions#
- 1 2 3 4 5 6 PC
51 nt
17

Figure S5

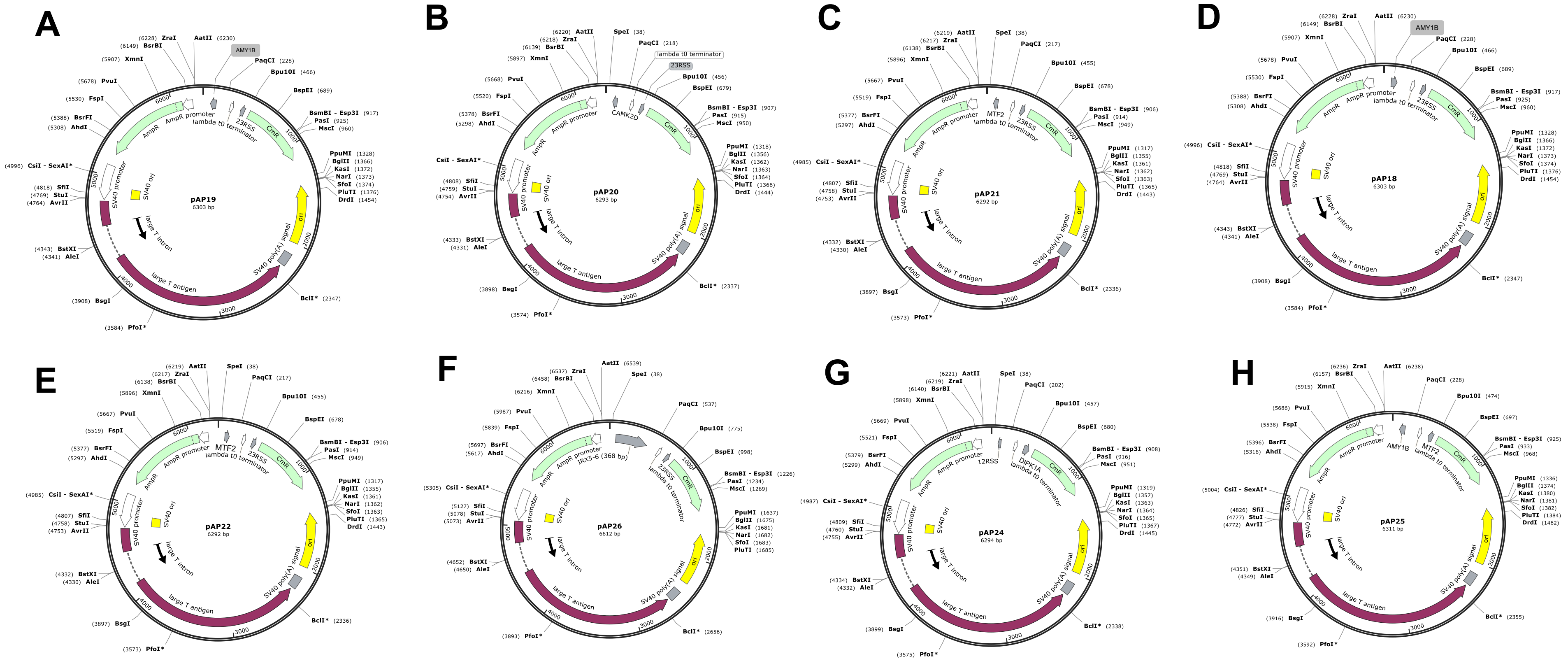
A
pAP19
6303 bp
B
pAP20
6293 bp
C
pAP21
6292 bp
D
pAP18
6303 bp
E
pAP22
6292 bp
F
pAP26
6612 bp
G
pAP24
6294 bp
H
pAP25
6311 bp

Figure S6

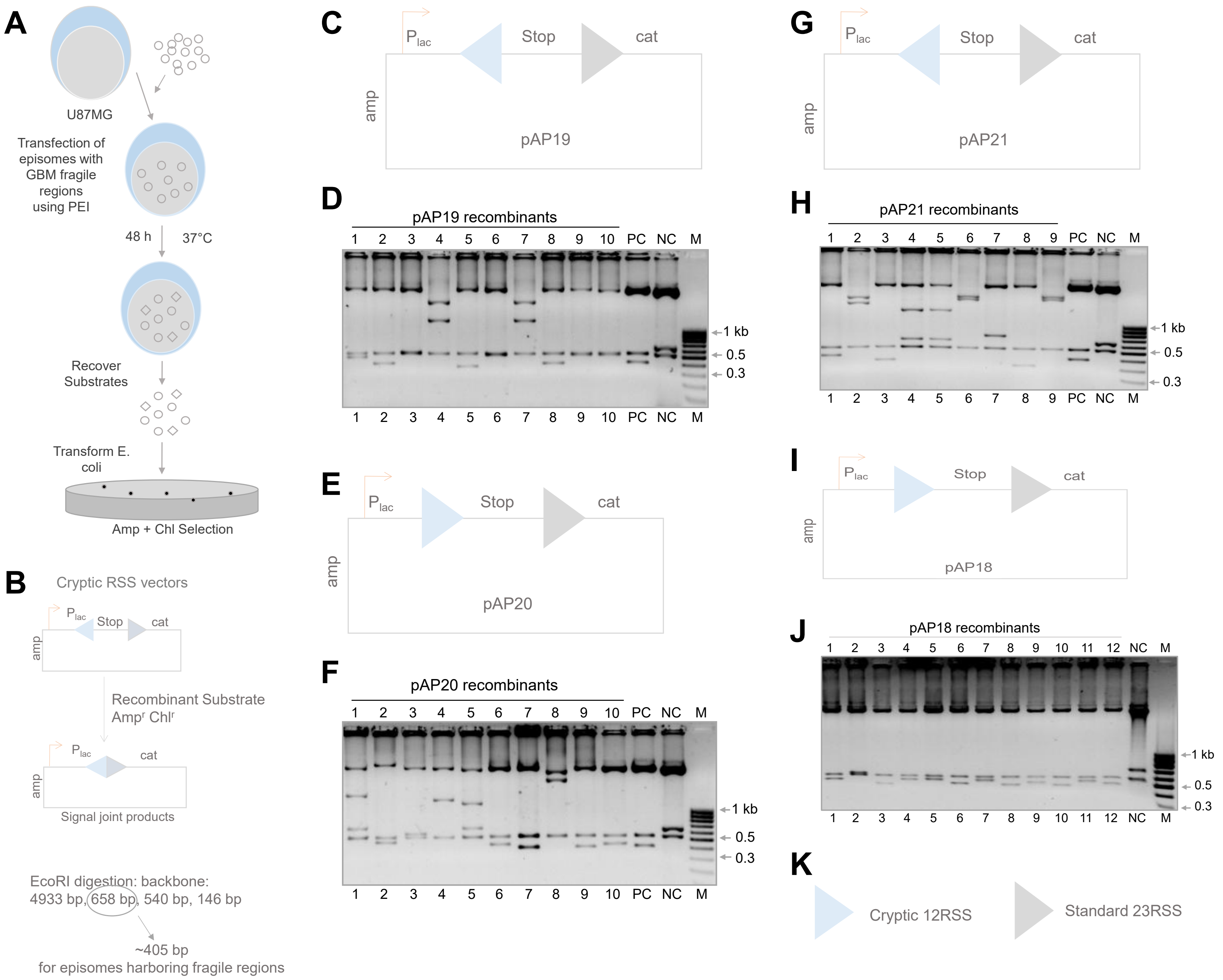
A
U87MG
Transfection of episomes with GBM fragile regions using PEI
48 h
37°C
Recover Substrates
Transform E. coli
Amp + Chl Selection
B
Cryptic RSS vectors
Plac
Stop
cat
amp
Recombinant Substrate
Ampr Chlr
Signal joint products
EcoRI digestion: backbone:
4933 bp, 658 bp, 540 bp, 146 bp
~405 bp
for episomes harboring fragile regions
C
pAP19
D
pAP19 recombinants
1 2 3 4 5 6 7 8 9 10 PC NC M
1 kb
0.5
0.3
E
pAP20
F
pAP20 recombinants
G
pAP21
H
pAP21 recombinants
1 2 3 4 5 6 7 8 9 PC NC M
I
pAP18
J
pAP18 recombinants
1 2 3 4 5 6 7 8 9 10 11 12 NC M
K
Cryptic 12RSS
Standard 23RSS

Figure S7

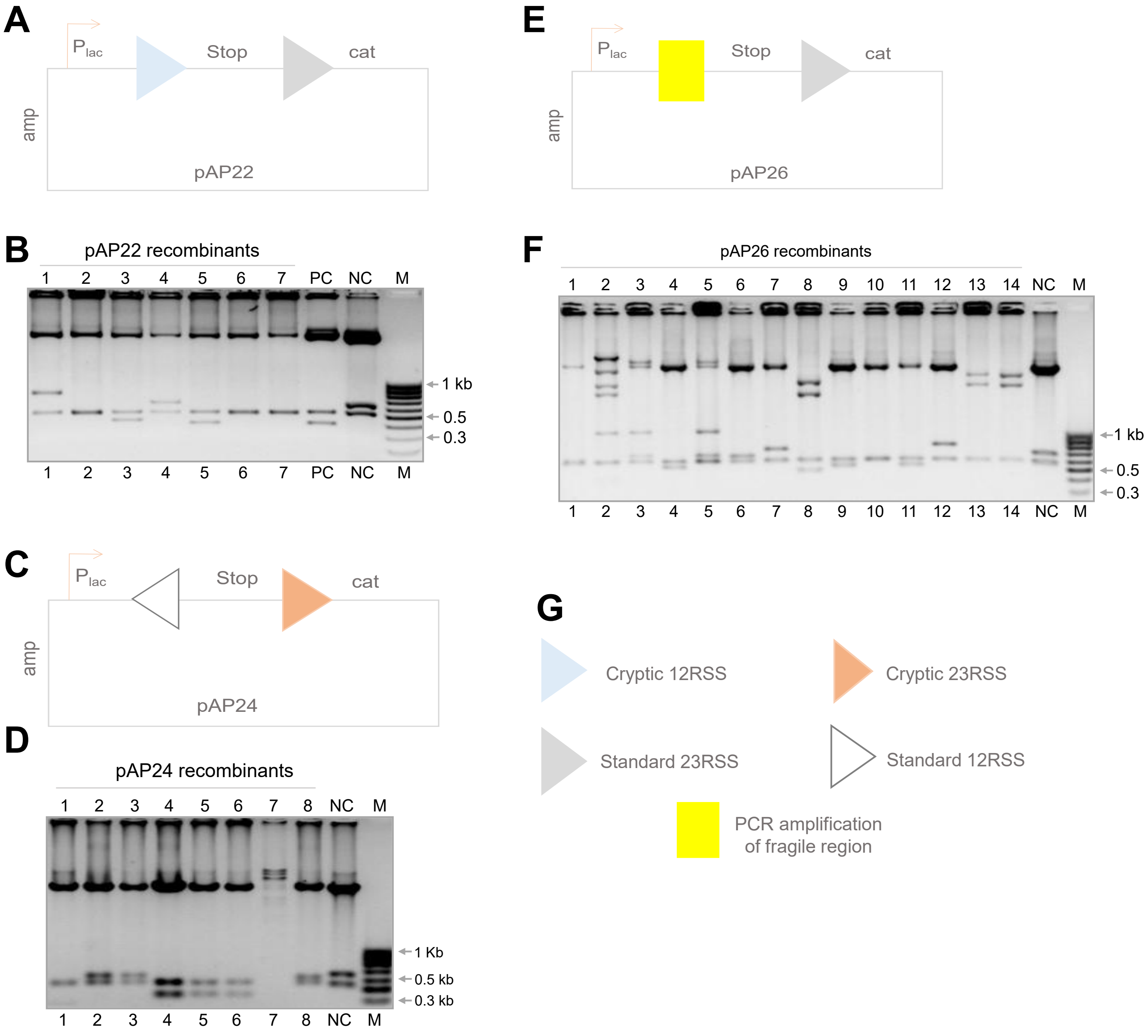
A
P lac
Stop
cat
amp
pAP22
B
pAP22 recombinants
1 2 3 4 5 6 7 PC NC M
1 kb
0.5
0.3
C
P lac
Stop
cat
amp
pAP24
D
pAP24 recombinants
1 2 3 4 5 6 7 8 NC M
1 Kb
0.5 kb
0.3 kb
E
P lac
Stop
cat
amp
pAP26
F
pAP26 recombinants
1 2 3 4 5 6 7 8 9 10 11 12 13 14 NC M
1 kb
0.5
0.3
G
Cryptic 12RSS
Cryptic 23RSS
Standard 23RSS
Standard 12RSS
PCR amplification of fragile region
